# Quercetin Preserves Oral Cavity Health by Mitigating Inflammation and Microbial Dysbiosis

**DOI:** 10.3389/fimmu.2021.774273

**Published:** 2021-11-26

**Authors:** Erin C. Mooney, Sara E. Holden, Xia-Juan Xia, Yajie Li, Min Jiang, Camille N. Banson, Bin Zhu, Sinem Esra Sahingur

**Affiliations:** ^1^ Department of Periodontics, School of Dental Medicine, University of Pennsylvania, Philadelphia, PA, United States; ^2^ Department of Human and Molecular Genetics, School of Medicine, Virginia Commonwealth University, Richmond, VA, United States; ^3^ Department of Periodontics, School of Dentistry, Virginia Commonwealth University, Richmond, VA, United States; ^4^ Department of Microbiology and Immunology, School of Medicine, Virginia Commonwealth University, Richmond, VA, United States

**Keywords:** flavonoids, periodontal diseases, IL-6, TNF, A20, microbiome, toll-like receptor (TLR), NF-κB

## Abstract

Failure to attenuate inflammation coupled with consequent microbiota changes drives the development of bone-destructive periodontitis. Quercetin, a plant-derived polyphenolic flavonoid, has been linked with health benefits in both humans and animals. Using a systematic approach, we investigated the effect of orally delivered Quercetin on host inflammatory response, oral microbial composition and periodontal disease phenotype. *In vivo*, quercetin supplementation diminished gingival cytokine expression, inflammatory cell infiltrate and alveolar bone loss. Microbiome analyses revealed a healthier oral microbial composition in Quercetin-treated versus vehicle-treated group characterized by reduction in the number of pathogenic species including *Enterococcus, Neisseria* and *Pseudomonas* and increase in the number of non-pathogenic *Streptococcus* sp. and bacterial diversity. *In vitro*, Quercetin diminished inflammatory cytokine production through modulating NF-κB:A20 axis in human macrophages following challenge with oral bacteria and TLR agonists. Collectively, our findings reveal that Quercetin supplement instigates a balanced periodontal tissue homeostasis through limiting inflammation and fostering an oral cavity microenvironment conducive of symbiotic microbiota associated with health. This proof of concept study provides key evidence for translational studies to improve overall health.

## Introduction

A symbiotic consortium between microbiome, microbiome- associated molecular patterns (MAMPs) and innate sensors such as Toll like receptors (TLR) are responsible for a regulated immune response and sustained periodontal tissue homeostasis. Ligand recognition by TLRs initiates an array of signaling cascades including nuclear factor kappa-light-chain-enhancer of activated B cells (NF-κB), one of the archetypical drivers of the host and adaptive immune response in periodontitis (1–3). The subsequent release of cytokines and chemokines is necessary for priming host immunity and maintaining periodontal tissue integrity. To avoid excessive inflammatory responses, host cells are equipped with negative-regulatory mechanisms to control TLR-mediated inflammatory responses and restore immune system balance. Ubiquitination is one of the most prominent regulator of signaling downstream of TLRs (4, 5). Ubiquitination is a highly dynamic, enzymatically-catalyzed posttranslational modification, which regulates cellular and immune functions through distinct polyubiquitin signals (6). To date, ubiquitination and ubiquitin-editing molecules have been characterized as important regulators of NF-κB in the pathophysiology of numerous chronic inflammatory conditions (7, 8). Ubiquitin-editing enzyme, A20, also known as TNF-α inducible protein 3 (TNFAIP3) has emerged as a critical gatekeeper of immune homeostasis in various systemic conditions, including periodontitis, through its ability to limit inflammation (9–12). If one or more of these regulatory mechanisms go awry and the initial immune response fails to terminate timely, the constant influx of inflammatory mediators creates a local microenvironment favorable for the growth of gram-negative, inflammogenic oral bacteria resulting in microbial dysbiosis (11, 13–16). The subsequent oral landscape promotes a continuous rhythmic process in which inflammation and dysbiosis collectively drive periodontal tissue destruction in susceptible individuals and lead to periodontal disease, one of the most common chronic diseases worldwide. Further, persistent chronic localized inflammation is associated with multiple systemic complications (1, 17–19). Therefore, the development of targeted strategies which regulate inflammation is critical for the maintenance of periodontal tissue homeostasis and overall health.

The discovery of natural compounds targeting the host immune responses offer promise to sustain health and improve clinical outcomes. Quercetin, a natural plant-derived dietary polyphenol, possesses high safety profile and extensive beneficial properties including potent antioxidant, anti-inflammatory, anti-cancer, antiviral, anti-hypertensive and anti-aging drug effects (20–26). Quercetin has been used to improve the disease outcomes in several disorders including rheumatoid arthritis, neuroinflammation and gastrointestinal disorders (27–30). Similarly, emerging evidence also indicates the oral-protective properties of Quercetin. Periodontal disease progression is driven by deregulated inflammation and a dysbiotic microbiota underlying the importance of evaluating the ability of therapeutic compounds to modulate both the host tissue responses and the microbiome. Yet, there are still no studies which assessed the effect of Quercetin supplementation on inflammation and the oral microbiome simultaneously in the course of periodontitis.

We report here the results of first systematic investigation which assessed the effect of oral delivery of Quercetin during the course of periodontitis through monitoring changes in inflammation, microbiome and alveolar bone loss. To accomplish this goal, periodontitis was induced in mice using ligatures to observe changes in the microbiota and the host without the addition of exogenous pathogens (31, 32). Using this *in vivo* model of periodontitis, we were able to show for the first time that oral delivery of Quercetin facilitates a sustained periodontal tissue homeostasis and mitigates the disease through modulating inflammatory response and oral microbial composition. Corroborating *in vivo* data, Quercetin diminished cytokine production through its effect on NF-κB:A20 signaling axis in human derived macrophage like cells exposed to lipopolysaccharide (LPS) and periodontal bacteria: *Porphyromonas gingivalis*, and *Fusobacterium nucleatum.* These preclinical studies reveal proof-of-concept evidence identifying Quercetin as a promising natural based therapeutic to restore periodontal-host and -microbiome tissue homeostasis.

## Materials and Methods

### Animals

10-12-week-old male C57BL/6J mice were purchased from Jackson Laboratories (Bar Harbor, Maine). All mice were maintained in a specific pathogen free (SPF) environment and housed in the same room. Mice were provided with water and a standard laboratory diet ad libitum. They were supplied with hardwood chips as bedding and housed in a temperature-controlled, air-conditioned room on a 12-hr light-dark cycle. For each independent experiment, 10 mice were assigned to the Quercetin treated group, while 5 mice were assigned to the vehicle treated group. All the mice were cohoused for two weeks to standardize microbial communities prior to the start of experiments (33). On day 1 of the study, mice were randomly assigned to experimental groups by husbandry staff with no involvement in the study design. Thereafter, mice were housed in separate cages based on experimental groups. Cages were changed daily to prevent Quercetin redosing by coprophagy and prevent feces accumulation and limit coprophagy (34, 35). Mice either received twice daily oral administration of Quercetin (40mg/kg/twice per day; Cayman Chemical: Item No.10005169) or an equal amount of phosphate buffered saline (PBS: Life Technologies) for 5 days. Body surface area was used to determine the concentration of Quercetin to be used in the mice. By using body surface area, a 40mg/kg dosage in mice would equate to 195mg of Quercetin for a 60kg human (36). Quercetin was solubilized in an ethanol-water mixture to enhance its absorption (37). A one-inch straight stainless-steel oral gavage needle with a round-ball stainless steel tip was placed at the end of a 1mL syringe and it was used for oral administration. Quercetin and vehicle were administered in small, 50µl, doses to make sure all the compound was swallowed before the introduction of the remaining medication. The total dosing volume was 300 µl of either Quercetin or vehicle two times daily. Periodontal inflammation and bone loss were induced by ligature placement on day 6 of the experimental timeline. Quercetin-treated and vehicle-treated were anesthetized intraperitoneally with a 200 µl mixture of ketamine (10mg/mL) and xylazine. Black braided 5-0 threads were placed at the left side of the maxilla interdentally between the first and second molars, while the right sides remained un-ligated as controls. Twice daily oral administration of Quercetin or vehicle treatment resumed on day 7 and mice were euthanized one-week post-ligation. Oral swabs of teeth and gingival tissue were taken before the induction of periodontitis on day 1 and day 6 and final swabs were taken at the completion of the study on day 13. The gingival tissues around all maxillary molars, without including palatal or buccal mucosa, were carefully excised using a new sterile #15 scalpel blade under 3.5x magnification by a trained periodontist using Dental Loupes (38–41). The mice were excluded from the study if the underlying bone was damaged during excision of the gingival tissues or the ligatures were lost. Maxillary jaws and gingival tissues were assessed for bone loss and inflammatory mediators and cellular infiltrate, respectively (42–44). The Institutional Animal Care and Use Committee of Virginia Commonwealth University approved all procedures and experiments were carried out following their guidelines.

### Microbes

The bacterial strains were *Porphyromonas gingivalis* (strain ATCC 33277) and *Fusobacterium nucleatum* (strain ATCC 25586) and grown anaerobically (5% CO_2_, 10% H_2_, 85% N_2_) at 37°C. *P. gingivalis* was maintained in Brain Heart Infusion broth supplemented with 0.05% Yeast Extract, 5g/mL Hemin, 0.5g/mL Vitamin K and 0.1% cysteine. *F. nucleatum* was grown in Brain Heart Infusion broth containing 5 mg/L Hemin, 0.5 mg/L menadione, 1g/L cysteine, 5g/L Yeast Extract, 0.001% *N-*acetyl muramic acid, and 5% fetal bovine serum. The bacterial suspensions were killed by heating at 80°C for 10 minutes. Sterility of killed bacteria was confirmed by enumeration of CFU on an agar plate.

### Cell Culture

The human monocytic leukemia cell line (THP-1; ATCC TiB-202) were grown in in RPMI 1640 (Life Technologies, Cat# 11875093) media supplemented with 10mM HEPES (Sigma, Cat# 1101100250), 1mM sodium pyruvate (Fisher Scientific, Cat# 11360070), 4500 mg/L glucose (Fisher Scientific, Cat# D16-500), 0.05 mM mercaptoethanol (Sigma, Cat# M6250-100ML) 10% fetal bovine serum, and 1% penicillin/streptomycin (Invitrogen, Cat# 15140122) at 37°C in a humidified 5% CO_2_ atmosphere. The cells were maintained in a logarithmic phase of growth (2 × 10^5^ to 8 × 10^5^) by passage every 3 to 4 days and were subsequently used for *in vitro* stimulation assays.

### Microscopic Computed Tomography Analysis

Maxillae were dissected after euthanasia and fixed in 10% neutral-buffered formalin for at least 24 hours prior to imaging and scanned with a desktop micro-CT system (Brüker Skyscan 1173, Skyscan NV, Kontich, Belgium) at a resolution of 1,120 × 1,120 pixels (image pixel size of 15.82 µm) over 180^o,^80 kV voltage, 80 µA current and 250 ms exposure time. Five x-ray projections were acquired every 0.2° and averaged. A standard Feldkamp reconstruction was done using NRecon software (Brüker) with a beam-hardening correction of 15% and a Gaussian-smoothing kernel of 1. Histomorphometric analysis was performed with the DataViewer MicroCT visualization Software (Brüker, Kontich, Belgium) with a 1,092 × 1,092 pixel size in all three spatial dimensions and setting the sagittal plane parallel to the X-ray beam axis. The amount of linear alveolar bone present was calculated and measured with DataViewer software using sagittal images measuring the distance from the cemental-enamel junction (CEJ) to the alveolar bone crest (ABC). Histogram settings were set to 255 to measure the CEJ-ABC junction distance and also to distinguish between hard tissues (i.e. alveolar bone) and enamel from the soft tissues. Linear measurements were taken (in millimeters) from the CEJ to the ABC in the interdental region between the first and second molars (M1-M2) or the second and third molars (M2-M3). All scans were reoriented with DataViewer to the same position for bone loss evaluation such that the CEJ and the root apex next to the measurement appeared in the micro-CT slice that was to be analyzed and standardize measurements according to previously established methods (44–51). Specifically, all images were oriented so that the CEJ of the first and second molar were parallel to the horizontal axis and the CEJ and root apex appeared in the same slice generating a standardized region of interest with set anatomical limits. A total of four CEJ-ABC measurements were made from the sagittal view in DataViewer. The average of the four linear measurements from each mouse was used for the subsequent analysis. Measurements were made by a blinded-investigator and analysis was performed three times for each mouse with similar results. Data are presented from 5 independent experiments from a total of 37 Quercetin-treated mice and 21 vehicle-treated control mice. One-Way ANOVA analysis using GraphPad Prism software from GraphPad Software Inc (La Jolla, CA, USA) was performed to compare the means of the 4 independent groups (Control+Vehicle, Ligature+Vehicle, Control+Quercetin, Ligature+Quercetin). 3D representative images were obtained using CTVox 3D visualization software (Brüker). Linear measurements were not made using this software as Brüker’s 3D visualization software solely offers 3D model creation, viewing and flexible control of Micro-CT scans (52–54).

### Histological Study of Mouse Gingival Tissues

Gingival tissues from Quercetin-treated and vehicle-treated control mice were removed using previously established protocols with slight modifications (44, 55). Using a #15 scalpel blade and 3.5x magnification Dental Loupes, a trained periodontitis excised the gingival tissues surrounding the three maxillary molars on the ligated or control side of the maxilla without including buccal and palatal tissues and damaging underlying tissues or bone (38–41). Tissues were fixed in 4% formaldehyde and sent to the Virginia Commonwealth University Cancer Mouse Models Core Laboratory where they were processed to paraffin. Briefly, fixed tissues were processed under a vacuum using the automated Tissue Tek Tissue Processor VIP (Sakura Finetek, Torrance, CA). The processor dehydrated the tissue using gradual increases in concentrations of ethanol from 70% to 100%, then was cleared with Citrisol (National Diagnostics) finishing with 4 changes of Paraplast Plus (VWR) paraffin wax at 60 degrees. Processed and paraffin embedded samples are then individually placed into a stainless steel mold containing molten Paraplast Plus with an embedding ring and allowed to harden on a cryo plate for 20 minutes before removing from mold. Using a standard rotary microtome, 5µm thick sections are floated on a 40-degree C water bath and then mounted onto positive charged slides and set on end to dry at room temperature. Progressive H&E staining of paraffin embedded sections is performed using the automated Agilent Dako CoverStrainer slide processing system. The instrument process involves baking, dewaxing, hydrating and staining through to the dehydrated, coverslipped and dried slide. Hematoxylin, Eosin and Bluing Buffer and mounting medium are purchased from Agilent Technologies as validated ready-to-use reagents for the Dako CoverStrainer. Histology images were acquired using the Q-Color 5 imaging system from Olympus Microscopy with a 10× magnification objective lens. Quantification of numbers of nucleated (hematoxylin-positive) in the gingiva was performed by a blinded examiner using CellSens software at 20× magnification. Counting was performed using the “Count and Measure” tool and the “Manual Threshold” option to choose an initial nucleated cell for reference. Once the cell was selected, subsequent cells were automatically selected until all nucleated cells in the connective tissue were highlighted. Data were reported as area stained (square micrometers) in each field of view. Nine regions/fields of view were analyzed per ligated side, and six regions/fields of view were analyzed per control side, per mouse, respectively. A minimum of 7 mice were examined per treatment group and data are representative of three independent analyses.

### Quantitative Real-Time Polymerase Chain Reaction

Gingival tissues from Quercetin-treated and vehicle-treated control mice were isolated and harvested using previously established protocols with slight modifications and as described in the previous sections (44, 55). Total RNA was extracted from gingival tissues using the RNeasy kit (Qiagen, Cat# 74136) and genomic DNA (gDNA) eliminator spin columns. The RNA concentrations were determined with NanoDrop. For each gingival side of each mouse approximately 1800 ng- 7500 ng of total RNA was isolated. Samples were never pooled of mice receiving the same treatment. 800 ng of total RNA was used for cDNA synthesis with High Capacity cDNA Reverse Transcription Kit, following the users’ manual (Applied Biosystems, Cat# 4368814). Primers were obtained by Invitrogen. Sequences for primers used were as follows: mIL-6 forward: 5′-TCTATACCACTTCACAAGTC GGA-3′, mIL-6 reverse: 5′-GAAT TGCCATTGCACAACTCTTT-3′; mTNF forward: 5′-CTGAACTTCGGGGTGAT CGG -3′, mTNF reverse: 5′-GGCTTGTCACTCGAATTTTGAGA-3′, TNFAIP3/A20 (mouse) forward: 5′-AGGTCGGTGTGAACGGATTTG -3′, and reverse: 5′-GGACAGTTGGGTGTCTCACATT-3′; TNFAIP3/A20 (human) forward: 5’-TTGTCCTCAGTTTCGGGAGAT-3’ and reverse: 5’-ACTTCTCGACACCAGTTGACTT-3’. Results were normalized with respect to the values obtained for the housekeeping gene GAPDH. ΔCt was calculated by subtracting the Ct value of the housekeeping gene (GAPDH) from the target gene Ct value. ΔΔCt was calculated by subtracting the control group target gene ΔCt from the experimental group target gene ΔCt. Relative mRNA expression was calculated by 2^-ΔΔCt^. Data are presented as averages of 3 independent experiments from 26 Quercetin-treated mice and 11 vehicle-treated control mice.

### Microbial Sample Processing and Library Preparation

Bacteria were obtained by oral swabbing of the teeth and gingival surface with Ultra-Fine polystyrene swab (Puritan Medical Products) and placed in DNA/RNA Shield (Zymo Research, Irvine, CA). Samples were set to ZymoBIOMICS for Targeted Metagenomic Sequencing. DNA Extraction was performed using ZymoBIOMICS-96 MagBeadDNA Kit (Zymo Research, Irvine, CA). The DNA samples were prepared for targeted sequencing with the *Quick-*16S NGS Library Prep Kit (Zymo Research, Irvine, CA). The primer sets used were *Quick-*16S Primer Set V3-V4 (Zymo Research, Irvine, CA). These primers were custom-designed by Zymo Research to provide the best coverage of the 16S gene while maintaining high sensitivity. The sequencing library was prepared using an innovative library preparation process in which PCR reactions were performed in real-time PCR machines to control cycles and therefore limit PCR chimera formation. The final PCR products were quantified with qPCR fluorescence readings and pooled together based on equal molarity. The final pooled library was cleaned up with the Select-a-Size DNA Clean & Concentrator (Zymo Research, Irvine, CA) then quantified with TapeStation (Agilent Technologies, Santa Clara, CA) and Qubit (Thermo Fisher Scientific, Waltham, WA). The ZymoBiomics Microbial Community Standard (Zymo Research, Irvine, CA) was used as a positive control for each DNA extraction and each targeted library preparation. Negative controls (blank extraction control, blank library preparation control) were included to assess the level of bioburden carried by the wet-lab process.

### Sequencing Data Analysis

The final library was sequenced on Illumina MiSeq with a v3 reagent kit (600 cycles). The sequencing was performed with >10% PhiX spike-in. The quality control, feature table construction, diversity and taxonomic analysis were performed in QIIME2. The t-Distributed Stochastic Neighbor Embedding (t-SNE) plot was created by Rtsne package and the differential abundance was tested by ALDEx2 package in R. The raw data with quality scores lower than 25 was trimmed in a quality control step. Alpha diversity was performed by testing the observed OTU, evenness and Shannon index. The significance of alpha diversity was analyzed using Kruskal–Wallis test and corrected using the Benjamini–Hochberg procedure with a false discovery rate of 5%. Beta diversity metrics was computed using Bray-Curtis distance, following by PERMANOVA statistical analysis and corrected using the Benjamini–Hochberg procedure with a false discovery rate of 5%. PERMANOVA is a multivariate ANOVA with permutations. It is meant to test differences between groups with a lot of variables and with permutations to avoid possible biases. Statistical differences reveal that the distribution and abundances of experimental groups are different (56). The ordination plot was generated by performing dimensionality reduction using t-SNE. Silva-132-99-nb-classifier was used to assign taxonomy to the OTUs, following by differential abundance analysis using LEfSe (57). The abundance of taxa was determined by dividing the hits of a specific taxon by the total hits of all taxa in the sample. Our analyses identified a total of 163 species which were present in levels greater than 0.1% in more than 5% of total samples. These bacteria were included for differential abundance analysis. Sequencing was performed in each individual sample once. Additionally, the OTUs were also assigned to taxonomy using HOMD 16s rRNA database, following by the same steps for differential abundance analysis.

### *In Vitro* Stimulation Assays

THP-1 cells were suspended at a density of 3 × 10^6^ in 6-well plates and treated with 25ng/mL of phorbol 12-myristate 13-acetate (PMA: Sigma, Cat# P1585) overnight to differentiate into macrophages (43, 58, 59) The cells were then treated with Quercetin (5µg/mL) dissolved in ethanol for 2 hours prior to stimulation with bacteria and toll-like receptor agonists. The cells were challenged with heat-killed *P.gingivalis* (multiplicity of infection [MOI] of 1:100), *F.nucleatum* (MOI 1:50), *P. gingivalis* lipopolysaccharide (LPS) (10µg/mL) (*In vivo*gen, Cat# tlrl-ppglps), Pam3Cys-Ser-(Lys)4 (Pam3CSK4) (10ng/mL)(*In vivo*gen, Cat# tlrl-pms), and CpG oligonucleotide (ODN) 2006 (100µg/mL)(*In vivo*gen, Cat# tlrl-2006-5) for up to 24 hours. Inflammatory cytokine levels (TNF and IL-6) were determined in cell-free culture supernatants using ELISA (TNF ELISA kit: Thermo Fisher, Cat# 88-7346-22 and IL-6 ELISA kit: Thermo Fisher, Cat# 88-7066-22). For *in vitro* assays evaluating A20 mRNA levels, the cells were challenged with heat-killed *F.nucleatum* (multiplicity of infection [MOI] of 1:10), and Pam3Cys-Ser-(Lys)4 (Pam3CSK4) (10ng/mL)(*In vivo*gen, Cat# tlrl-pms) for up to 6 hours. Total RNA was isolated using RNeasy plus Mini Kit by QiaCube (Qiagen) and 800ng of total RNA was used for cDNA synthesis and subsequent qRT-PCR. All *in vitro* studies were performed with at least three sets of independent experiments with a minimum of 3 replicates per time point/stimulation.

### Immunofluorescence Staining

THP-1 cells (3x10^4^) were grown on 8-chamber polystyrene vessel tissue culture treated glass slide (BD Falcon, USA) and pretreated with 25ng/ml PMA to differentiate to macrophages overnight. The cells were then treated with Quercetin (5µg/mL) dissolved in ethanol for 2 hours prior to stimulation with bacteria and toll-like receptor agonists. The cells were stimulated with heat-killed *P.gingivalis* (multiplicity of infection [MOI] of 1:100), and *F.nucleatum* (MOI 1:50), for up to 60 minutes. Following treatment, cells were washed in cold PBS and then fixed in 4% paraformaldehyde. The cell membrane was then permeabilized in buffer containing 0.3% Triton-100 and 0.1% NaN3 in PBS for 30min at RT. Nonspecific binding sites were blocked by incubation with 5% BSA, 0.1% NaN3 and 0.3% triton-X in PBS in a humidity chamber for 1h at RT or overnight at 4°C. Cells were incubated with anti-NF-κBp65 antibody (ProteinTech, 1:100) overnight at 4°C. After incubation, cells were rinsed twice for 5 min in PBS with gentle shaking and incubated with Alexa 488 goat anti-rabbit polyclonal antibody (Invitrogen, 1:250) for 1h at RT. Cells were washed 6 times in PBS with gentle shaking. Coverslips were mounted with antifade mounting medium with DAPI (Vectashield Vibrance) and kept under dark conditions at RT. The images were collected by Nikon confocal laser microscope at a magnification of 630X and zoom 2 to assess nuclear localization of NF-κB. For each group a minimum of 4 images were collected. Three sets of independent experiments were conducted with a minimum of 120 cells analyzed.

### Statistical Analysis

Statistical analysis of microbial samples is given in the Sequencing Data Analysis section above. Statistical analysis for all other experiments was performed using GraphPad Prism software from GraphPad Software Inc (La Jolla, CA, USA). All data were expressed as the mean ± the standard deviation. The difference between two groups was established by the unpaired *t* test with Mann-Whitney correction. Multiple group comparisons were performed by one-way ANOVA with Tukey’s *post hoc* test to identify differences between specific groups. A value of p<0.05 was considered to be statistically significant. The number of animals examined per group and number of times the experiments were carried out are given above and in the Figure Legends.

## Results

### Quercetin Diminishes Gingival Inflammation and Alveolar Bone Loss in Experimental Periodontitis

**Figure 1:**
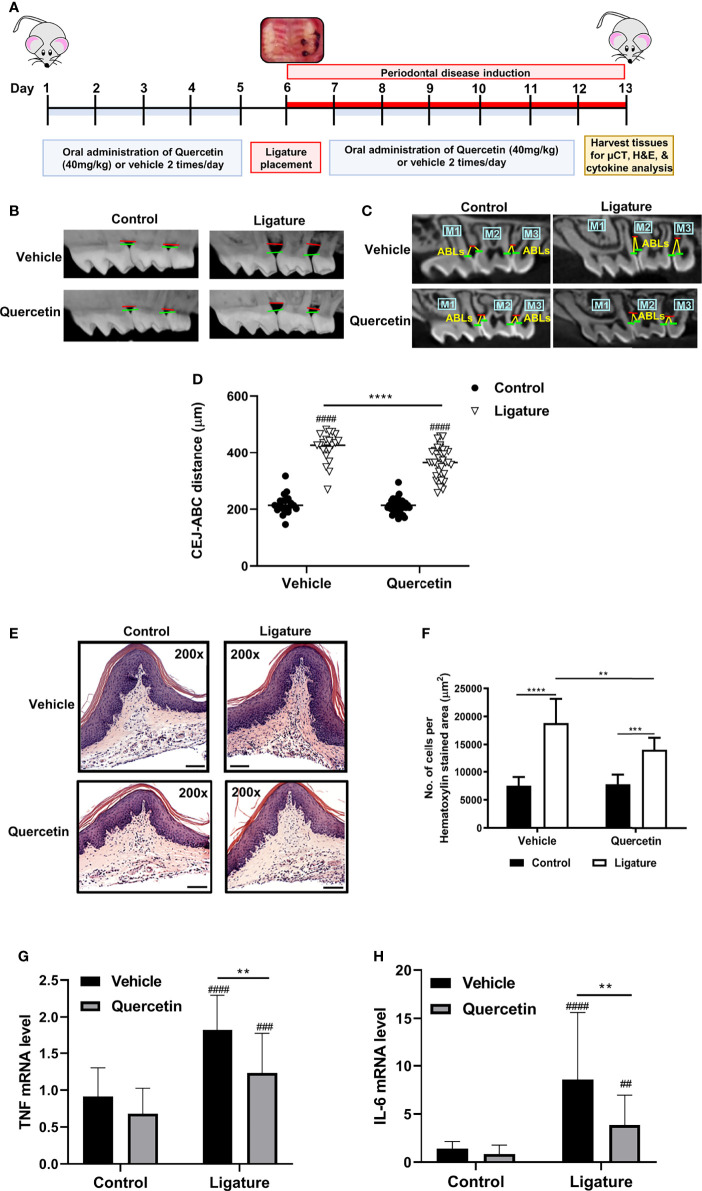
Quercetin Treatment Decreases Periodontal Inflammation and Bone Loss. **(A)** Schematic presentation demonstrating the experimental design system. In male C57BL/6J mice, silk 5-0 ligatures were placed on the left side of the maxilla interdentally between first and second molars, while the right sides were left unligated as controls. Mice were treated with Quercetin by oral gavage or vehicle matched control six days prior to ligature placement and maintained with the same treatment regimen one-week post-ligation. Mice were euthanized and periodontal tissues were examined. **(B)** Representative images of 3D micro-CT reconstructions of periodontal bone in Quercetin treated and vehicle treated mice. The distance between horizontal lines represents the distance between cemental-enamel junction and alveolar bone crest. **(C)** Representative two-dimensional images of sagittal slice views of Quercetin-treated and vehicle-treated mice. Linear measurements were taken of the alveolar bone loss (ABL) in the interdental space from the cemental-enamel junction to alveolar bone crest. **(D)** Distance between the cemental-enamel junction and alveolar bone crest was analyzed and plotted to determine periodontal bone loss (n=37 mice for Quercetin treatment, n=21 mice for vehicle treatment). The average of 4 CEJ-ABC measurements were calculated per mouse. Individual mouse datapoints are plotted. Averages and standard deviations are shown. ****p ≤ 0.0001. ^####^Ligated gingival tissues versus corresponding experimental controls (p ≤ 0.0001). **(E)** Representative images from H&E stained histological sections from gingival tissues derived from ligated and control periodontium in Quercetin-treated and vehicle-treated mice. **(F)** Quantitative analysis of inflammatory cell infiltrate in the periodontium. Positive cells were counted per µm^2^ gingival connective tissue. Data are representative of three independent experiments, with a minimum of seven mice analyzed in each treatment group per experiment. Averages and standard deviations are shown. **p ≤ 0.01, ****p ≤ 0.0001. **(G, H)** Gingival tissues derived from ligated and control periodontium in Quercetin treated and vehicle treated mice were digested and applied to RNA preparation. The mRNA levels of mTNF **(G)**, mIL-6 **(H)** were determined with qRT-PCR and the relative mRNA expression levels were plotted (n=11 for vehicle treatment, n=26 for Quercetin treatment). Averages and standard deviations are shown. **p ≤ 0.01, ***p<0.001, ****p ≤ 0.0001. ^####^Ligated gingival tissues versus corresponding experimental controls (p ≤ 0.0001). ^###^Ligated gingival tissues versus corresponding experimental controls (p ≤ 0.001). ^##^Ligated gingival tissues versus corresponding experimental controls (p ≤ 0.01).

The efficacy of Quercetin to modulate the immune responses and periodontal disease phenotype was assessed using the murine ligature-induced periodontitis model. Quercetin was administered orally at a dosage (40mg/kg/twice daily) comparable to humans a week before ligations and continued throughout the experimental period as described (**Figure 1A**). As anticipated, alveolar bone loss was observed in both groups following ligations (**Figures 1B, C**). Supporting a protective effect, the linear bone loss in the distance from the cementoenamel junction and alveolar bone crest was reduced in mice which received oral Quercetin supplement compared to the control group (**Figure 1D**). Periodontal bone loss typically mirrors the degree of inflammation present, therefore we further assessed immune cell infiltration in the dissected gingival tissues using H&E staining. In corroboration with the bone loss data, ligated sites displayed increased inflammatory cell infiltration in both experimental and control groups whereas the immune cell infiltrate in the ligated tissues of Quercetin-supplemented mice was significantly reduced compared to that of the mice in the vehicle group (**Figures 1E, F**). We subsequently determined gingival tissue cytokine expression to further elucidate the effect of Quercetin on the inflammatory response in the oral mucosa. As expected, induction of periodontitis led to the increased TNF and IL-6 expression in the gingival tissues. Substantiating the efficacy of oral delivery of Quercetin and anti-inflammatory action, the gingival tissues dissected from Quercetin-supplemented mice displayed significantly diminished cytokine expression profiles compared with their vehicle-treated counterparts (**Figures 1G, H**).

### Quercetin Mitigates Oral Microbial Dysbiosis in Experimental Periodontitis

**Figure 2:**
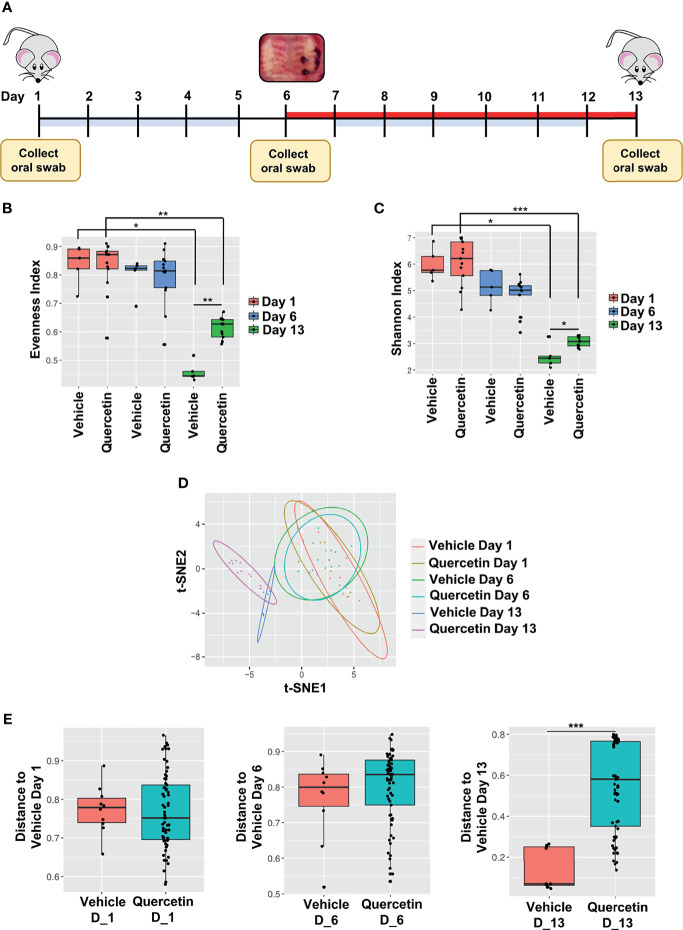
Quercetin Treatment Increases Bacterial Diversity in Mice with Experimental Periodontitis. **(A)** Schematic diagram illustrating the periodontal induction and treatment experimental design and timeline of oral swab collection for subsequent 16S rRNA sequencing microbiome analysis. **(B, C)** Alpha diversity was assessed in the oral microbiota of 5 vehicle- and 13 Quercetin-treated mice before and after the induction of periodontitis and the Evenness **(B)** and Shannon index **(C)** of vehicle- and Quercetin-treated mice on Day 1, 6 and 13 are shown. *p ≤ 0.05, **p ≤ 0.01. **(D, E)** Dissimilarity was assessed in the oral microbiota of 5 vehicle- and 13 Quercetin-treated mice before and after periodontitis induction and the ordination is shown by the t-Distributed Stochastic Neighbor Embedding (t-SNE) plot **(D)**. The Bray-Curtis dissimilarity between vehicle- and Quercetin-treated mice in Days 1 (left), 6 (middle) and 13 (right) are shown **(E)**. ***p ≤ 0.001.

**Figure 3:**
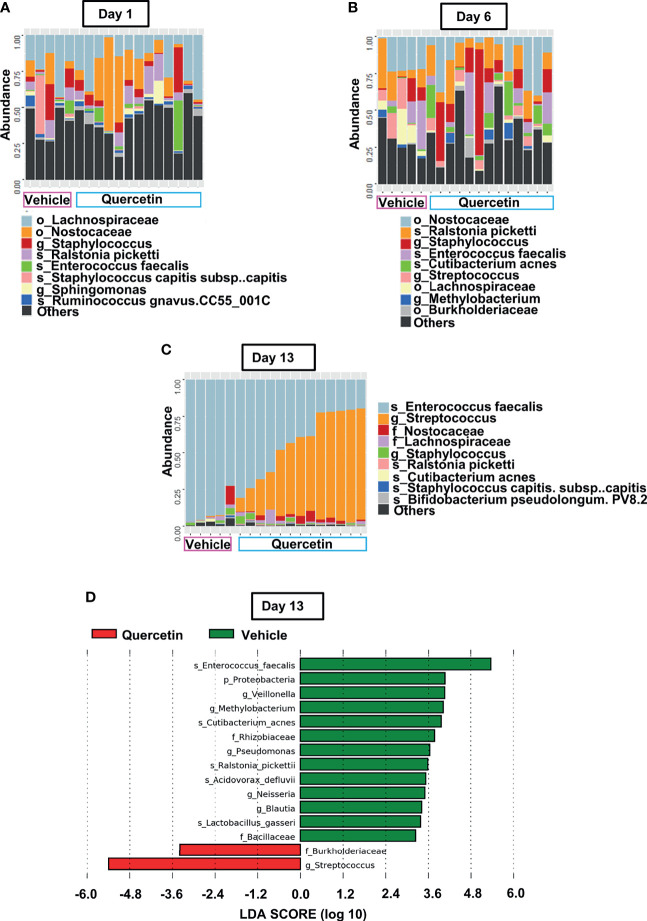
Quercetin Treatment Alters Microbial Composition Induced by Periodontitis. **(A–C)** Prominent bacterial taxa in 5 vehicle- and 13 Quercetin-treated mice at Day 1 **(A)**, Day 6 **(B)** and Day 13 **(C)**. **(D)** Significantly altered taxa in Quercetin treated compared to vehicle treated mice after periodontitis onset (Day 13) as determined by linear discriminant analysis effect size (LDA) scores using Silva-132 classifier.

To characterize Quercetin effects on the temporal dynamics of inflammation-induced microbial dysbiosis within the oral cavity, changes in the oral microbiota were assessed upon treatment with Quercetin or vehicle matched control before and after periodontitis onset as described in **Figure 2A**. Oral bacteria were collected at the baseline of the study (Day 1), time of ligature placement (Day 6), and after the disease onset (Day 13). Before the onset of periodontitis (Days 1 and 6), the oral microbiota of each treatment group displayed increased alpha diversity which is consistent with healthy microbiota compared to those collected following disease establishment (Day 13) (**Figures 2B, C**). After periodontitis onset, the Evenness index was decreased compared to healthy group indicating few taxonomic groups dominate during the disease (**Figure 2B**). Similar results were obtained with the Shannon index, reflecting less community richness and evenness in the oral microbiota of animals driven by experimental periodontitis (**Figure 2C**). Confirming the efficacy of oral Quercetin delivery, after periodontitis onset, Quercetin-supplemented mice exhibited significantly higher Evenness and Shannon indices relative to the vehicle-treated group (**Figures 2B, C**). These results suggest that Quercetin supplement aids to maintain the diversity of the oral microbiome. The taxonomic compositions of each treatment group were also distinguished by dissimilarity (**Figures 2D, E**). Through visualization of clusters using the t-SNE algorithm, two-dimensional ordination demonstrated similarity of the oral microbiome between the treatment groups prior to periodontitis induction (**Figure 2D**). However, after periodontitis onset, treatment groups were well-separated indicating heterogeneity between the oral microbial composition of Quercetin and vehicle treated mice (**Figure 2D**). Variation in community composition among treatment groups was also characterized by Bray-Curtis measures of taxonomic distance (**Figure 2E**). As expected, significance was only observed in the microbial composition between Quercetin and vehicle-treated mice after the induction of periodontitis (**Figure 2E**). Corroborating our alpha and beta diversity analyses, the mice from both treatment groups displayed similar taxonomic profiles before the development of periodontitis (**Figures 3A, B**). However, after periodontitis onset, there were several species-, genus-, and family- level differences between bacterial communities in Quercetin-treated and vehicle-treated mice (**Figure 3C**). Mice which received Quercetin had increased levels of *Streptococcus*, which is often associated with a symbiotic microbiome (**Figure 3D**). Additionally, mice which received vehicle treatment, displayed increased levels of several bacteria including *Enterococcus, Neisseria* and *Pseudomonas*, which are often associated with periodontitis or other inflammatory conditions (**Figure 3D**). Furthermore, when using HOMD reference database specific for the oral microbiome, Quercetin-treated mice displayed increases in *Streptococcus sanguinis* and *Streptococcus parasanguinis*, known commensal bacteria in the oral cavity (**Supplementary Figure 1**).

### Quercetin Maintains Periodontal Tissue Homeostasis Through its Effect on NF-κB:A20 Axis and Cytokine Production

**Figure 4:**
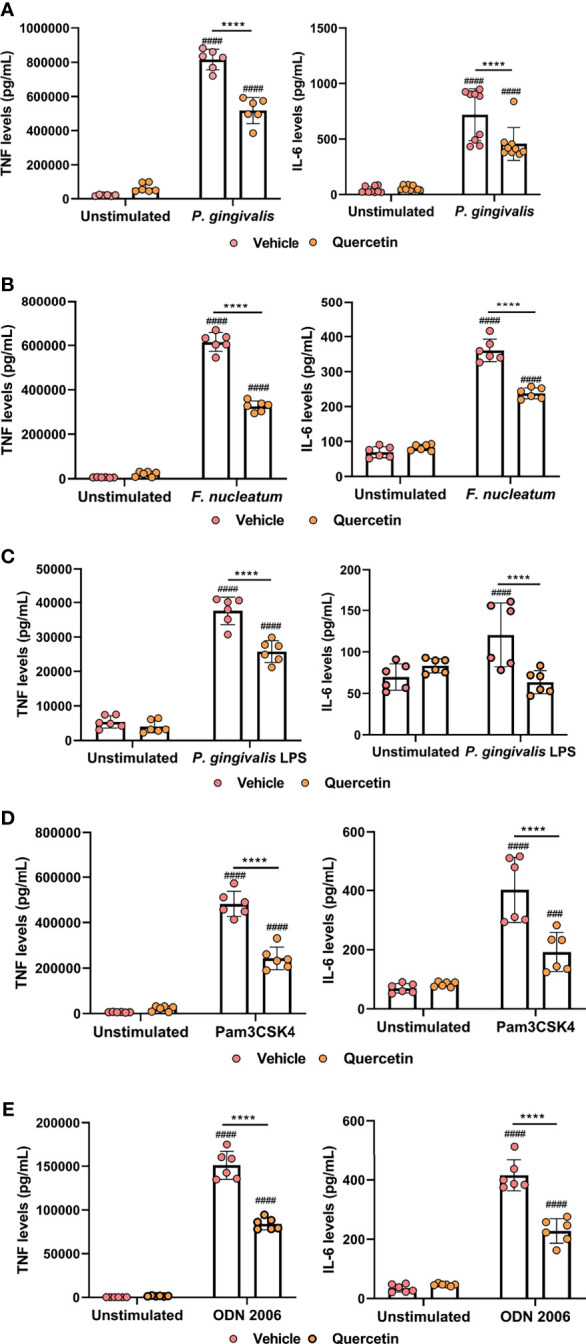
Quercetin diminishes cytokine production in human macrophages challenged with oral bacteria and toll-like receptor agonists. THP-1 macrophages treated with Quercetin (5µg/mL) or vehicle matched control were infected with oral bacteria or toll-like receptor agonists for up to 24 hours. **(A–E)** IL-6 and TNF levels in the supernatants were determined with ELISA. ELISA was conducted on **(A)** macrophages infected with *P. gingivalis* (100 MOI), **(B)** macrophages infected with *F*. *nucleatum* (50 MOI), **(C)** macrophages infected with *P. gingivalis* LPS (10µg/mL), **(D)** macrophages infected with Pam3CSK4 (10ng/mL) and **(E)** macrophages infected with ODN 2006 (100µg/mL). Each experiment was performed at minimum three times independently and averages and standard deviations are shown. ****p ≤ 0.0001. ^####^Unstimulated control cells versus corresponding stimulated cells (p ≤ 0.0001). ^###^Unstimulated control cells versus corresponding stimulated cells (p ≤ 0.001).

The interaction between host immune cells and the oral microbiome, and subsequent crosstalk between innate signaling pathways play a critical role in determining periodontal disease outcome. Macrophages are one of the key cells responding to microbial insult in periodontitis pathogenesis. We therefore sought to determine the effect of Quercetin on TLR signaling and cytokine responses in human macrophages challenged with periodontal bacteria and various TLR agonists. *P. gingivalis* and *F. nucleatum* were included as representative organisms as they possess unique virulence factors favoring microbial dysbiosis and periodontal tissue destruction and are frequently associated with numerous systemic complications (60–63). Briefly, macrophages were treated with Quercetin for 2 hours prior to challenge with bacteria or TLR agonist, LPS, and cytokine response was assessed post-infection. Corroborating its anti-inflammatory action, Quercetin treatment significantly reduced TNF and IL-6 production in human macrophages following infection with *P. gingivalis* (**Figure 4A** and **Supplementary Figure 2A**) and *F. nucleatum* (**Figure 4B** and **Supplementary Figure 2B**). Cytokine levels remained comparable in Quercetin-treated unstimulated control macrophages compared to vehicle-treated unstimulated control cells, suggesting that Quercetin functions to mitigate inflammation downstream of TLR signaling. To further characterize the effect of Quercetin on inflammatory response, macrophages were treated with Quercetin or vehicle and challenged with TLR agonists ([*P. gingivalis* LPS for TLR4], [Pam3CSK4 for TLR2], and [CpG oligonucleotide 2006 [ODN] for TLR9]) for up to 24 hours (**Figures 4C–E** and **Supplementary Figures 2C-E**). As expected, quercetin treated cells produced significantly less cytokine in response to *P. gingivalis* LPS (**Figure 4C** and **Supplementary Figure 2C**), Pam3CSK4 (**Figure 4D** and **Supplementary Figure 2D**) and ODN2006 (**Figure 4E** and **Supplementary Figure 2E**) compared to vehicle-treated group.

**Figure 5:**
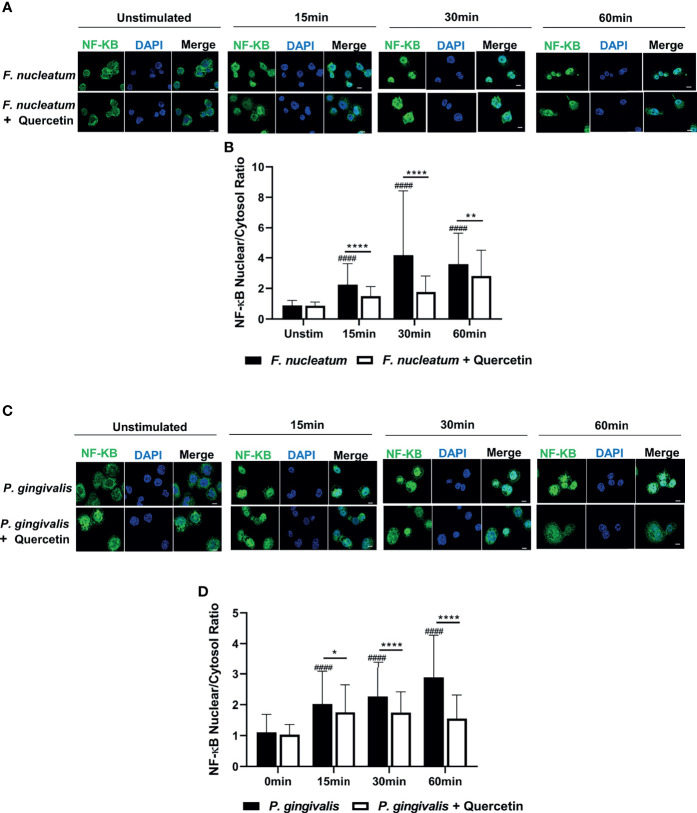
Quercetin decreases NF-κB nuclear translocation upon LPS-and oral bacteria-induced inflammation in human macrophages. THP-1 macrophages treated with Quercetin (5µg/mL) or vehicle matched control were infected with LPS or oral bacteria for indicated times. NF-κB was stained with NF-κB antibody followed by Alexa-Fluor488 (green) and the nucleus was stained with DAPI (blue). Images were captured with Nikon Confocal laser microscope. **(A)** Representative images of vehicle- and Quercetin-treated cells infected with *F. nucleatum* (25 MOI) **(C)** and *P. gingivalis* (50MOI) and the quantitative analysis of nuclear to cytosolic ratio of NF-κB was shown, respectively **(B, D)**. Scale bars=10µM. Data are representative of three independent experiments and averages and standard deviations are shown. *p ≤ 0.05, **p ≤ 0.01, ****p ≤ 0.0001. ^####^Unstimulated control cells versus corresponding stimulated cells (p ≤ 0.0001).

**Figure 6:**
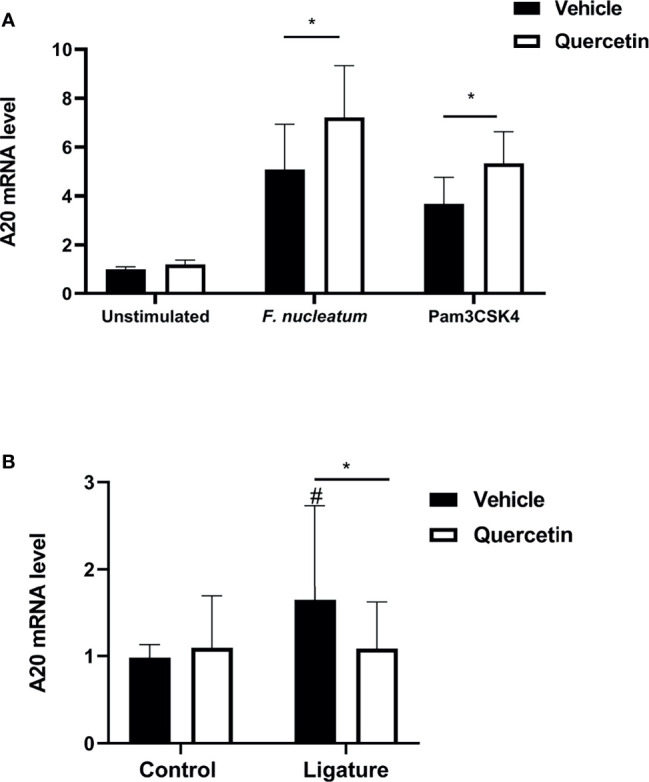
Quercetin modulates A20 mRNA levels in pre-clinical periodontitis disease models. **(A)** THP-1 macrophages treated with Quercetin (5µg/mL) or vehicle matched control were infected with oral bacteria (*F.nucleatum* [10 MOI]) or toll-like receptor agonist (Pam3CSK4 [10ng/mL]) for up to 6 hours and applied to RNA preparation. The mRNA levels of mA20 were determined with qRT-PCR and the relative mRNA expression levels were plotted. Each experiment was performed three times independently and averages and standard deviations are shown. *p ≤ 0.05. **(B)** Gingival tissues derived from ligated and control periodontium in Quercetin treated and vehicle treated mice were digested and applied to RNA preparation. The mRNA levels of mA20 were determined with qRT-PCR and the relative mRNA expression levels were plotted (n=11 for vehicle treatment, n=26 for Quercetin treatment). Averages and standard deviations are shown. *p ≤ 0.05. ^#^Ligated gingival tissues versus corresponding experimental controls.

We then sought to determine the mechanisms that Quercetin can exert its effect on periodontal inflammation and define downstream signaling pathways. The TLR : NF-κB:A20 signaling axis is one of the critical pathways governing inflammation in the oral mucosa through modulating cytokine gene expression. We first assessed NF-κB nuclear translocation, a hallmark of NF-κB activation, in vehicle- and Quercetin-treated human macrophages using immunofluorescence and confocal imaging. Our results showed increased NF-κB translocation in vehicle-treated macrophages upon infection with *F. nucleatum* (**Figures 5A, B**) and *P. gingivalis* (**Figures 5C, D**) compared to Quercetin-treated cells indicating that Quercetin regulates oral bacteria-induced inflammatory cytokine response through its effect on NF-κB signaling pathway. The ubiquitin-editing enzyme, A20, is an inducible and broadly expressed cytoplasmic protein that inhibits TNF- and TLR-induced NF- κB activity and previous studies suggested its modulation by Quercetin treatment (9, 10, 64, 65). To determine the effect of Quercetin on A20 expression in the oral mucosa, we next examined A20 mRNA levels in response to Quercetin treatment in human macrophage-like cells challenged with *F. nucleatum-* and Pam3CSK4 and noted increased A20 expression in cells treated with Quercetin compared to those which received vehicle (**Figure 6A**). Further, we also assessed the effect of Quercetin on gingival tissue A20 expression *in vivo*. As expected, there was an upregulation of A20 mRNA in the ligated gingival tissues of vehicle treated mice, indicating enhanced inflammation in these tissues (**Figure 6B**). Consistent with the improved disease phenotype, the ligated tissues of Quercetin treated mice displayed similar A20 mRNA levels compared to the control tissues (**Figure 6B**). Overall, these results support *in vivo* data and confirm the anti-inflammatory effect of Quercetin as it applies to host-microbiome interactions in the oral cavity. It is also of note that consistent with the proven clinical safety profile of Quercetin, we observed no effect on cell viability or structure throughout the experiments which indicates that decreased cytokine production was not due to undesirable effects of Quercetin on macrophages.

## Discussion

**Figure 7:**
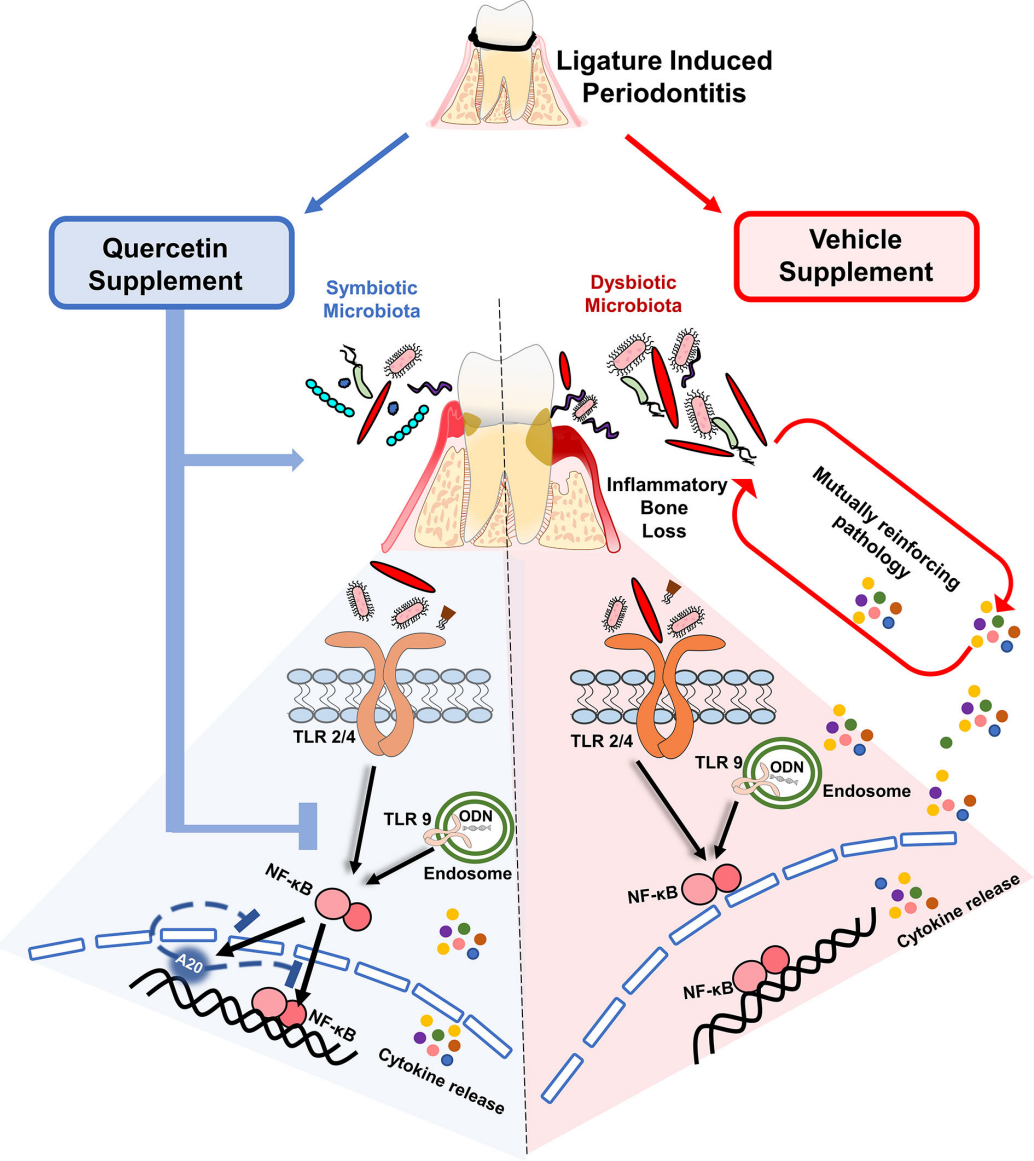
Quercetin supplement alleviates the progression of periodontitis by aiding in the preservation of host- and microbiome-homeostasis. A self-perpetuating pathogenic cycle of deregulated inflammation, exacerbated cytokine release, and bacterial dysbiosis fuels tissue destruction and the subsequent development of periodontitis in response to ligature placement. Quercetin, a ubiquitous polyphenolic flavonoid, promotes health through various biological mechanisms. Upon periodontitis induction, Quercetin supplement reduced gingival inflammation, alveolar bone loss and limits bacterial dysbiosis. At the cellular level, periodontal tissue homeostasis requires the stringent regulation of inflammatory pathways mainly driven by the activation of innate sensors, such as TLRs. Downstream of TLR activation, Quercetin supplement decreased cytokine production by regulating NF-κB activation and possibly modulating A20 expression, in response to oral bacteria and TLR agonist challenge. Collectively, the regulation of inflammation with Quercetin supplement has a significant impact on gingival inflammatory gene expression and bacterial composition that is coincident with ameliorating disease progression.

Despite many advances in the field, periodontal diseases continue to be one of the most common inflammatory conditions worldwide posing a significant overall health problem and financial burden (66, 67). There is an urgent need to develop effective, safe, cheap and practical preventive approaches to facilitate the maintenance of a balanced host-microbiome interactions and prevent the progression of the disease. This is especially important for susceptible populations such as aging, diabetics, and immunocompromised, who exhibit severe forms of the diseases (68–71). In this investigation, we report key data for future translational studies which show that oral Quercetin usage can improve periodontal disease outcomes through its effect on host inflammatory response and oral microflora (**Figure 7**). Specifically, we noted significantly improved disease phenotype as measured by decreased alveolar bone loss, inflammatory cell infiltrate, and gingival tissue cytokine expression in Quercetin-treated versus vehicle-treated mice. We further confirmed the mechanism of anti-inflammatory action of Quercetin using an *in vitro* disease model and revealed that Quercetin can diminish inflammatory response to periodontal bacteria and in human macrophage-like cells through its effect on NF-κB signaling pathway.

The oral cavity is in constant exposure to microbial and physical insult and therefore it is crucial to identify novel ways to mitigate the effect of these stressors to sustain periodontal tissue homeostasis and prevent disease progression. The current paradigm for the treatment of periodontal disease supports development of novel strategies which target key molecular pathways to limit prolong inflammation and facilitate timely resolution (72). Recently, research efforts focus on harnessing the beneficial properties of natural products and mediators in periodontal inflammation as safe and cost-effective therapeutics (31). In general, the studies which investigate the efficacy of immune modulatory reagents administer compounds after the disease induction phase to evaluate therapeutic effects and mostly use local injections as the delivery method to achieve maximum concentration at the lesion site (31, 34, 73). In this study we introduced Quercetin through an oral route prior to the onset of the disease in an attempt to enhance absorption into periodontal tissues and establish a sustained effect (74, 75). In addition, using this study design we were able to evaluate the effect of this natural compound both in health and disease states. Indeed, Quercetin had no impact on oral microbial composition in health as shown by the lack of differences in the microbiome between Day 1 and Day 6 of the study whereas it improved dysbiosis during the course of the disease (Day 13). These results further confirm the safety profile, efficacy and health benefits of Quercetin in the oral cavity. Our results concur with previous studies which delivered Quercetin through subcutaneous and intragastric delivery to halt periodontitis and reveal for the first time the effectiveness of oral delivery which is a more clinically plausible and practical application for supplemental use in periodontal practice (76–78). In fact, after just 2 weeks of Quercetin supplementation, mice exhibited decreased alveolar bone loss compared to vehicle-treated mice. Most recently, Quercetin was shown to prevent oxidative stress-induced injury of periodontal ligament cells and reduce alveolar bone loss in periodontitis after intragastric delivery (78). Similar beneficial effects of Quercetin have been consistently noted in other immune and inflammatory conditions which share common pathophysiological features with periodontitis (28–30, 79–82). *In vitro* studies involving human and mouse cells as well as *in vivo* mouse models have detailed the role of Quercetin in protecting against neuroinflammation by inhibiting nitric oxide production and neuronal apoptosis and prevent inflammation-related neuronal injury and neurodegeneration (83–86). It has been reported to improve disease phenotype in collagen- and zymosan-induced arthritis in mice by decreasing inflammatory cytokine levels, cartilage and bone destruction, and synovial inflammation as well (87, 88). During experimental allergic inflammation in mice, orally administered Quercetin significantly reduced cytokine levels in bronchoalveolar lavage fluid and mucus production in the lungs (89). Similarly, Quercetin supplementation has been demonstrated to decrease susceptibility for the development of asthma, bronchial hyper-reactivity and chronic obstructive pulmonary diseases in human and mouse studies (90–93). Quercetin also has been reported to improve diabetes-related complications in *in vivo* mouse and rat studies by interfering with the innate signaling pathways such as TLRs and peroxisome proliferator-activated receptor ɣ (PPARɣ) and inhibiting the activity of nuclear factor κ light-chain enhancer of activated B cells (NF-κB) which subsequently reduces the levels of TNF and CRP (94–96). As therapies targeting the modulation of the host immune response continue to offer promise alleviating adverse clinical outcomes, these results collectively suggest that Quercetin supplementation may be effective to sustain health at both oral and distant sites. In light of our current findings and accumulating evidence supporting the benefits of Quercetin in health and disease, future studies are warranted to develop local delivery methods and assess the efficacy using different concentrations in the management of periodontal diseases in a more targeted fashion before and after the onset of the disease (31). Considering the established associations between the severity of periodontitis and numerous systemic conditions including diabetes, it would be also of interest to determine whether Quercetin supplementation can improve periodontal clinical parameters in these susceptible patient cohorts as well.

In the oral cavity, the initiation of inflammation is largely driven by engagement of the microbiome and microbiome associated molecular patterns with TLRs and the subsequent activation of NF-κB and inflammatory mediator production. Hitherto, there was a lack of knowledge about the mechanistic role of Quercetin in the oral cavity at the cell-microbiome interface. Previous studies have investigated the potent anti-inflammatory effects of Quercetin in human gingival fibroblasts, a cell type predominating in gingival connective tissue. Quercetin was shown to inhibit the inflammatory response to *P. gingivalis* LPS in human gingival fibroblasts *via* suppressing the NF-κB signaling pathway (97). In early periodontal lesions, macrophages also play a critical role in the immune landscape. Macrophages counteract pressures from a diversity of stimuli, including the oral microbiome, which can drive their polarization into distinct inflammatory or resolution states (98, 99). Therefore, understanding how therapeutic agents drive these responses will be critical for elucidating determinants of successful resolution in periodontitis. In this study, our results indicated that Quercetin functions as a regulator of inflammation through modulating NF-κB and cytokine production in macrophages upon oral microbial infection. These findings are consistent with previous mechanistic studies which revealed that Quercetin attenuates inflammation through interfering with multiple signaling pathways including NF-κB, mitogen-activated protein kinase (MAPK), phosphatidylinositol-3-kinase/Akt (PI3K/Akt), signal transducer and activator of transcription proteins (STAT), inflammasome complex and regulates cellular functions including apoptosis, cellular senescence, and autophagy in several models of diseases and cell types (100–108). Notably, the beneficial role of Quercetin has also been extensively studied in cancer, and the potent anti-inflammatory and anti-tumor functions have been documented in breast cancer and liver cancer through its ability to inhibit NF-κB signaling cascade (109, 110). One of the key negative regulators of NF-κB activation is the ubiquitination enzyme, A20 (9–11, 111). In several inflammatory diseases, including periodontal disease, A20 plays role as one of the key regulatory agents (43, 44, 64). Recent evidence suggests that Quercetin can diminish inflammation through its effect on A20 in cystic fibrosis cells (65). Similarly, we were able to show the induction of A20 in response to Quercetin treatment in *F. nucleatum-* and Pam3CSK4-treated human macrophage-like cells. This observation is consistent with the previous reports and our current data demonstrating reduced NF-κB activity following Quercetin treatment *in vitro* and suggests that Quercetin can regulate inflammation through the NF-κB/A20 signaling axis. Further, we also assessed the effect of Quercetin on gingival tissue A20 expression *in vivo*. A20 is a downstream regulator of NF-κB and its levels are closely regulated by the level of inflammation. As expected, there was increased A20 expression in the ligated sites of vehicle treated mice compared to unligated sites at Day 13 which indicate increased levels of inflammation. In contrast, ligated sites of Quercetin treated mice displayed similar level of A20 expression as the healthy (unligated) sites which provides further evidence that Quercetin treatment alleviates inflammatory response in the periodontal tissues. Recent studies have also suggested Quercetin’s mode of action is mediated by several factors including transcriptional, posttranscriptional and posttranslational regulation through direct DNA binding, microRNA regulation, and modulation of epigenetic machinery such as DNA methyltransferase and histone deacetylase activities (112–115). In the current study, *in vivo* disease model was originally designed to assess the effect of Quercetin on periodontitis phenotype and gingival biopsies were harvested at the end of the experimental period. Endogenous regulators of inflammation are dynamic and their levels are tightly regulated in the tissues to sustain tissue homeostasis. To fully characterize the mechanism of action of Quercetin on different signaling pathways and monitor dynamic changes *in vivo*, future studies are warranted using biological specimens obtained at multiple time points during the initiation, progression and resolution phases of the disease. Overall, considering how genetic and epigenetic alterations affect the progression of periodontitis and associated conditions, new insights into mechanisms by which Quercetin influences cellular functions can pave the way to understanding and regulating its action in targeted therapies in humans.

The hallmark of advanced periodontal lesion is impaired host-microbiome homeostasis which is characterized by prolonged inflammation, decreased polymicrobial diversity and further tissue damage (13–15). In addition to determining the effect of Quercetin on periodontal inflammation, we also monitored changes in oral microbiome composition before and after disease induction. As expected, there was decreased microbial diversity in all groups of mice with periodontitis compared to healthy groups. When comparisons were performed among periodontitis groups, mice receiving Quercetin supplements displayed increased bacterial diversity compared to the vehicle treated group. Consistently, species specific analyses revealed an increase in commensal flora, *S. sanguinis* and *S. parasanguinis*, after periodontitis onset in Quercetin treated group versus vehicle group (116). These results suggest that Quercetin treatment may support a periodontal tissue microenvironment which consists of a microbial composition associated with health. Further supporting this notion, vehicle-treated mice with periodontitis exhibited increases in *Enterococcus*, *Blautia* and *Lactobacillus*, which are all concomitant with periodontitis and periodontitis-related complications including diabetes (31, 34). Collectively, these results indicate that Quercetin treatment likely drives resilience against inflammophilic bacterial infection and was more effective at enriching beneficial oral taxa compared to vehicle treatment. Our results are also consistent with the studies reporting similar findings in the gut. Quercetin was shown to increase gut microbiome diversity and reduce the populations of *Fusobacterium* and *Enterococcus* in mice with inflammatory bowel disease protecting them against *C. rodentium*-induced colitis (80). These observations may have significant implications as *F. nucleatum* has emerged as an opportunistic pathogen in both oral and extraoral tissues and is associated with several disease outcomes (60, 62, 117). Remarkably, the therapeutic potential of Quercetin in remodeling gut microbiota has also been indicated in metabolic disorders such as obesity, nonalcoholic fatty liver disease, atherosclerosis, and diabetes-related sequelae such as diabetic peripheral neuropathy (118–121). While there is still no consensus whether Quercetin acts as an antimicrobial agent, it is likely that Quercetin-induced microbial changes are due to its effect on reducing inflammation (77). In fact, using a murine model of periodontitis induced by *Aggregatibacter actinomycetemcomitans* infection, subcutaneous treatment of Quercetin reduced bone loss and inflammatory cytokine production without affecting bacterial load on the last day of the experiment (77). Our study reveals that Quercetin does not appear to reshape the microbiome in states of health, as there were no observed differences between the microbiome in Day 1 and Day 6 of Quercetin- and vehicle-treated mice. Only after the induction of periodontitis were there differences in the microbiota, possibly due to Quercetin primarily modulating inflammatory pathways. Considering the diversity of the oral microbiome, it will certainly be crucial to determine how Quercetin can affect bacterial growth and virulence.

In summary, our data explicitly revealed that oral delivery of Quercetin helps sustain periodontal tissue health through mitigating inflammation and promoting a symbiotic microbial community composition (**Figure 7**). Due to its high tolerability and safety profile, it may prove as an effective, low-cost and long-term supplement in chronic disease populations, including those who are susceptible for periodontal diseases (21, 122–126).

## Data Availability Statement

The datasets presented in this study can be found in online repositories. The names of the repository/repositories and accession number(s) can be found below: NCBI; PRJNA776284.

## Ethics Statement

The animal study was reviewed and approved by The Institutional Animal Care and Use Committee of Virginia Commonwealth University.

## Author Contributions

SS contributed to the conception of the experiments. SS, EM, and SH contributed to the design of the experiments. EM, SH, X-JX, YL, MJ, and CB performed experiments. SS, EM, X-JX, YL, MJ, and BZ analyzed results. SS and EM wrote the manuscript. All authors contributed to the article and approved the submitted version.

## Funding

This work was supported by US Public Health Service grants R01DE025037 and R01DE027374 to SE. Sahingur from the National Institute of Dental and Craniofacial Research/National Institutes of Health.

## Conflict of Interest

The authors declare that the research was conducted in the absence of any commercial or financial relationships that could be construed as a potential conflict of interest.

## Publisher’s Note

All claims expressed in this article are solely those of the authors and do not necessarily represent those of their affiliated organizations, or those of the publisher, the editors and the reviewers. Any product that may be evaluated in this article, or claim that may be made by its manufacturer, is not guaranteed or endorsed by the publisher.

## Supplementary Material

Supplementary Figure 1 Quercetin Treatment Alters Oral Microbial Composition Induced by Periodontitis. Significantly altered taxa in Quercetin treated compared to vehicle treated mice after periodontitis onset (Day 13) as determined by linear discriminant analysis effect size (LDA) scores using HOMD 16S rRNA database.

Supplementary Figure 2 Quercetin diminishes cytokine production in human macrophages challenged with oral bacteria and toll-like receptor agonists. THP-1 macrophages treated with Quercetin (5µg/mL) or vehicle matched control were infected with oral bacteria or toll-like receptor agonists for up to 12 hours. **(A–E)** IL-6 and TNF levels in the supernatants were determined with ELISA. ELISA was conducted on **(A)** macrophages infected with P. gingivalis (100 MOI), **(B)** macrophages infected with F. nucleatum (50 MOI), **(C)** macrophages infected with P. gingivalis LPS (10µg/mL), **(D)** macrophages infected with Pam3CSK4 (10ng/mL) and **(E)** macrophages infected with ODN 2006 (100µg/mL). Each experiment was performed three times independently and averages and standard deviations are shown. ****p<0.0001. ####Unstimulated control cells *versus* corresponding stimulated cells (p<0.0001). #Unstimulated control cells *versus* corresponding stimulated cells (p<0.05).

## References

[1] Crump KE Sahingur SE. Microbial Nucleic Acid Sensing in Oral and Systemic Diseases. J Dent Res (2016) 95(1):17–25. doi: 10.1177/0022034515609062 26438211 PMC4700663

[2] Song B Zhang YL Chen LJ Zhou T Huang WK Zhou X. The Role of Toll-Like Receptors in Periodontitis. Oral Dis (2017) 23(2):168–80. doi: 10.1111/odi.12468 26923115

[3] Gu Y Han X. Toll-Like Receptor Signaling and Immune Regulatory Lymphocytes in Periodontal Disease. Int J Mol Sci (2020) 21(9):3329. doi: 10.3390/ijms21093329 PMC7247565 32397173

[4] Hu H Sun SC. Ubiquitin Signaling in Immune Responses. Cell Res (2016) 26(4):457–83. doi: 10.1038/cr.2016.40 PMC4822134 27012466

[5] Swatek KN Komander D. Ubiquitin Modifications. Cell Res (2016) 26(4):399–422. doi: 10.1038/cr.2016.39 27012465 PMC4822133

[6] Kliza K Husnjak K. Resolving the Complexity of Ubiquitin Networks. Front Mol Biosci (2020) 7(21). doi: 10.3389/fmolb.2020.00021 PMC7056813 32175328

[7] Rape M. Ubiquitylation at the Crossroads of Development and Disease. Nat Rev Mol Cell Biol (2018) 19(1):59–70. doi: 10.1038/nrm.2017.83 28928488

[8] Cockram PE Kist M Prakash S Chen S-H Wertz IE Vucic D. Ubiquitination in the Regulation of Inflammatory Cell Death and Cancer. Cell Death Differ (2021) 28(2):591–605. doi: 10.1038/s41418-020-00708-5 33432113 PMC7798376

[9] Wertz IE O’Rourke KM Zhou H Eby M Aravind L Seshagiri S. De-Ubiquitination and Ubiquitin Ligase Domains of A20 Downregulate NF-κb Signalling. Nature (2004) 430(7000):694–9. doi: 10.1038/nature02794 15258597

[10] Lee EG Boone DL Chai S Libby SL Chien M Lodolce JP. Failure to Regulate TNF-Induced NF-kappaB and Cell Death Responses in A20-Deficient Mice. Sci (New York NY) (2000) 289(5488):2350–4. doi: 10.1126/science.289.5488.2350 PMC3582399 11009421

[11] Mooney EC Sahingur SE. The Ubiquitin System and A20: Implications in Health and Disease. J Dental Res (2020) 100(1):10–20. doi: 10.1177/0022034520949486 PMC7755949 32853526

[12] Malynn BA Ma A. A20: A Multifunctional Tool for Regulating Immunity and Preventing Disease. Cell Immunol (2019) 340:103914. doi: 10.1016/j.cellimm.2019.04.002 31030956 PMC6584049

[13] Van Dyke TE Bartold PM Reynolds EC. The Nexus Between Periodontal Inflammation and Dysbiosis. Front Immunol (2020) 11:511–1. doi: 10.3389/fimmu.2020.00511 PMC7136396 32296429

[14] Curtis MA Diaz PI Van Dyke TE. The Role of the Microbiota in Periodontal Disease. Periodontol 2000 (2020) . 83(1):14–25. doi: 10.1111/prd.12296 32385883

[15] Frias-Lopez J Duran-Pinedo AE. The Function of the Oral Microbiome in Health and Disease. In: Sahingur SE, editor. Emerging Therapies in Periodontics. Cham: Springer International Publishing (2020). p. 141–73.

[16] Belibasakis GN Bostanci N Marsh PD Zaura E. Applications of the Oral Microbiome in Personalized Dentistry. Arch Oral Biol (2019) 104:7–12. doi: 10.1016/j.archoralbio.2019.05.023 31153099

[17] Beck JD Papapanou PN Philips KH Offenbacher S. Periodontal Medicine: 100 Years of Progress. J Dental Res (2019) 98(10):1053–62. doi: 10.1177/0022034519846113 31429666

[18] Konkel JE O’Boyle C Krishnan S. Distal Consequences of Oral Inflammation. Front Immunol (2019) 10:1403. doi: 10.3389/fimmu.2019.01403 31293577 PMC6603141

[19] Genco RJ Borgnakke WS. Risk Factors for Periodontal Disease. Periodontology (2000) 2013 62(1):59–94. doi: 10.1111/j.1600-0757.2012.00457.x 23574464

[20] Serban MC Sahebkar A Zanchetti A Mikhailidis DP Howard G Antal D. Effects of Quercetin on Blood Pressure: A Systematic Review and Meta-Analysis of Randomized Controlled Trials. J Am Heart Assoc (2016) 5(7):e002713. doi: 10.1161/JAHA.115.002713 27405810 PMC5015358

[21] Li Y Yao J Han C Yang J Chaudhry MT Wang S. Quercetin, Inflammation and Immunity. Nutrients (2016) 8(3):167. doi: 10.3390/nu8030167 26999194 PMC4808895

[22] Colunga Biancatelli RML Berrill M Catravas JD Marik PE. Quercetin and Vitamin C: An Experimental, Synergistic Therapy for the Prevention and Treatment of SARS-CoV-2 Related Disease (COVID-19). Front Immunol (2020) 11(1451). doi: 10.3389/fimmu.2020.01451 PMC7318306 32636851

[23] Carullo G Cappello AR Frattaruolo L Badolato M Armentano B Aiello F. Quercetin and Derivatives: Useful Tools in Inflammation and Pain Management. Future Med Chem (2016) 9(1):79–93. doi: 10.4155/fmc-2016-0186 27995808

[24] Mohammadi-Sartang M Mazloom Z Sherafatmanesh S Ghorbani M Firoozi D. Effects of Supplementation With Quercetin on Plasma C-Reactive Protein Concentrations: A Systematic Review and Meta-Analysis of Randomized Controlled Trials. Eur J Clin Nutr (2017) 71(9):1033–9. doi: 10.1038/ejcn.2017.55 28537580

[25] Vafadar A Shabaninejad Z Movahedpour A Fallahi F Taghavipour M Ghasemi Y. Quercetin and Cancer: New Insights Into its Therapeutic Effects on Ovarian Cancer Cells. Cell Biosci (2020) 10(1):32. doi: 10.1186/s13578-020-00397-0 32175075 PMC7063794

[26] Alam S Sarker MMR Afrin S Richi FT Zhao C Zhou J-R. Traditional Herbal Medicines, Bioactive Metabolites, and Plant Products Against COVID-19: Update on Clinical Trials and Mechanism of Actions. Front Pharmacol (2021) 12:671498–8. doi: 10.3389/fphar.2021.671498 PMC8194295 34122096

[27] Corsale I Carrieri P Martellucci J Piccolomini A Verre L Rigutini M. Flavonoid Mixture (Diosmin, Troxerutin, Rutin, Hesperidin, Quercetin) in the Treatment of I–III Degree Hemorroidal Disease: A Double-Blind Multicenter Prospective Comparative Study. Int J Colorectal Dis (2018) 33(11):1595–600. doi: 10.1007/s00384-018-3102-y 29934701

[28] Javadi F Ahmadzadeh A Eghtesadi S Aryaeian N Zabihiyeganeh M Rahimi Foroushani A. The Effect of Quercetin on Inflammatory Factors and Clinical Symptoms in Women With Rheumatoid Arthritis: A Double-Blind, Randomized Controlled Trial. J Am Coll Nutr (2017) 36(1):9–15. doi: 10.1080/07315724.2016.1140093 27710596

[29] Khan H Ullah H Aschner M Cheang WS Akkol EK. Neuroprotective Effects of Quercetin in Alzheimer’s Disease. Biomolecules (2019) 10(1):59. doi: 10.3390/biom10010059 PMC7023116 31905923

[30] Eid HM Haddad PS. The Antidiabetic Potential of Quercetin: Underlying Mechanisms. Curr Med Chem (2016) 24(4):355–64. doi: 10.2174/0929867323666160909153707 27633685

[31] Lee C-T Teles R Kantarci A Chen T McCafferty J Starr JR. Resolvin E1 Reverses Experimental Periodontitis and Dysbiosis. J Immunol (Baltimore Md 1950) (2016) 197(7):2796–806. doi: 10.4049/jimmunol.1600859 PMC5026932 27543615

[32] Graves DT Fine D Teng YT Van Dyke TE Hajishengallis G. The Use of Rodent Models to Investigate Host-Bacteria Interactions Related to Periodontal Diseases. J Clin Periodontol (2008) 35(2):89–105. doi: 10.1111/j.1600-051X.2007.01172.x 18199146 PMC2649707

[33] Witjes VM Boleij A Halffman W. Reducing Versus Embracing Variation as Strategies for Reproducibility: The Microbiome of Laboratory Mice. Anim (Basel) (2020) 10(12):2415. doi: 10.3390/ani10122415 PMC7767075 33348632

[34] Xiao E Mattos M Vieira GHA Chen S Correa JD Wu Y. Diabetes Enhances IL-17 Expression and Alters the Oral Microbiome to Increase Its Pathogenicity. Cell Host Microbe (2017) 22(1):120–128 e4. doi: 10.1016/j.chom.2017.06.014 28704648 PMC5701758

[35] Ye X Itzoe M Sarabia-Estrada R DeTolla L Tyler BM Guarnieri M. Suspected Lonely Mouse Syndrome as a Cage Effect in a Drug Safety Study. J Vet Med (2018) 2018:9562803–9562803. doi: 10.1155/2018/9562803 29854826 PMC5966667

[36] Reagan-Shaw S Nihal M Ahmad N. Dose Translation From Animal to Human Studies Revisited. FASEB J (2008) 22(3):659–61. doi: 10.1096/fj.07-9574LSF 17942826

[37] Azuma K Ippoushi K Ito H Higashio H Terao J. Combination of Lipids and Emulsifiers Enhances the Absorption of Orally Administered Quercetin in Rats. J Agric Food Chem (2002) 50(6):1706–12. doi: 10.1021/jf0112421 11879062

[38] Rios HF Giannobile WV. Preclinical Protocols for Periodontal Regeneration. In: Osteology Guidelines for Oral and Maxillofacial Regeneration: Preclinical Models for Translational Research. London; Chicago: Quintessence Publishing (2011). p. 77–102. Chapter 7.

[39] Marchesan J Girnary MS Jing L Miao MZ Zhang S Sun L. An Experimental Murine Model to Study Periodontitis. Nat Protoc (2018) 13(10):2247–67. doi: 10.1038/s41596-018-0035-4 PMC6773250 30218100

[40] Alvarez C Abdalla H Sulliman S Rojas P Wu Y-C Almarhoumi R. RvE1 Impacts the Gingival Inflammatory Infiltrate by Inhibiting the T Cell Response in Experimental Periodontitis. Front Immunol (2021) 12(1547). doi: 10.3389/fimmu.2021.664756 PMC8126725 34012448

[41] Liu J Chanumolu SK Krei Z Albahrani M Akhtam A Jia Z. Identification of Genes Differentially Expressed in Simvastatin-Induced Alveolar Bone Formation. JBMR Plus (2019) 3(5):e10122. doi: 10.1002/jbm4.10122 31131344 PMC6524672

[42] Lam RS O’Brien-Simpson NM Lenzo JC Holden JA Brammar GC Walsh KA. Macrophage Depletion Abates Porphyromonas Gingivalis–Induced Alveolar Bone Resorption in Mice. J Immunol (2014) 193(5):2349–62. doi: 10.4049/jimmunol.1400853 25070844

[43] Li Y Mooney EC Holden SE Xia XJ Cohen DJ Walsh SW. A20 Orchestrates Inflammatory Response in the Oral Mucosa Through Restraining NF-kappaB Activity. J Immunol (2019) 202(7):2044–56. doi: 10.4049/jimmunol.1801286 PMC6420508 30760622

[44] Crump KE Oakley JC Xia-Juan X Madu TC Devaki S Mooney EC. Interplay of Toll-Like Receptor 9, Myeloid Cells, and Deubiquitinase A20 in Periodontal Inflammation. Infect Immun (2017) 85(1):e00814–16. doi: 10.1128/IAI.00814-16 PMC5203663 27849177

[45] Park CH Abramson ZR Taba M Jr. Jin Q Chang J Kreider JM. Three-Dimensional Micro-Computed Tomographic Imaging of Alveolar Bone in Experimental Bone Loss or Repair. J Periodontol (2007) 78(2):273–81. doi: 10.1902/jop.2007.060252 PMC2581750 17274716

[46] Boyer E Leroyer P Malherbe L Fong SB Loréal O Bonnaure Mallet M. Oral Dysbiosis Induced by Porphyromonas Gingivalis is Strain-Dependent in Mice. J Oral Microbiol (2020) 12(1):1832837–1832837. doi: 10.1080/20002297.2020.1832837 33133418 PMC7580739

[47] Cho Y-J Song HY Ben Amara H Choi B-K Eunju R Cho Y-A. In Vivo Inhibition of Porphyromonas Gingivalis Growth and Prevention of Periodontitis With Quorum-Sensing Inhibitors. J Periodontol (2016) 87(9):1075–82. doi: 10.1902/jop.2016.160070 27177290

[48] Zhuang Z Yoshizawa-Smith S Glowacki A Maltos K Pacheco C Shehabeldin M. Induction of M2 Macrophages Prevents Bone Loss in Murine Periodontitis Models. J Dental Res (2019) 98(2):200–8. doi: 10.1177/0022034518805984 PMC6761736 30392438

[49] Pathak JL Fang Y Chen Y Ye Z Guo X Yan Y. Downregulation of Macrophage-Specific Act-1 Intensifies Periodontitis and Alveolar Bone Loss Possibly via TNF/NF-κb Signaling. Front Cell Dev Biol (2021) 9(474). doi: 10.3389/fcell.2021.628139 PMC7969798 33748112

[50] Gully N Bright R Marino V Marchant C Cantley M Haynes D. Porphyromonas Gingivalis Peptidylarginine Deiminase, a Key Contributor in the Pathogenesis of Experimental Periodontal Disease and Experimental Arthritis. PloS One (2014) 9(6):e100838. doi: 10.1371/journal.pone.0100838 24959715 PMC4069180

[51] Alvarez C Suliman S Almarhoumi R Vega ME Rojas C Monasterio G. Regulatory T Cell Phenotype and Anti-Osteoclastogenic Function in Experimental Periodontitis. Sci Rep (2020) 10(1):19018. doi: 10.1038/s41598-020-76038-w 33149125 PMC7642388

[52] Haugen HJ Qasim SB Matinlinna JP Vallittu P Nogueira LP. Nano-CT as Tool for Characterization of Dental Resin Composites. Sci Rep (2020) 10(1):15520. doi: 10.1038/s41598-020-72599-y 32968120 PMC7511412

[53] He L Xiao J Song L Zhou R Rong Z He W. HVEM Promotes the Osteogenesis of Allo-MSCs by Inhibiting the Secretion of IL-17 and IFN-γ in Vγ4t Cells. Front Immunol (2021) 12(2380). doi: 10.3389/fimmu.2021.689269 PMC8261146 34248977

[54] Huang Y Liao Y Luo B Li L Zhang Y Yan F. Non-Surgical Periodontal Treatment Restored the Gut Microbiota and Intestinal Barrier in Apolipoprotein E–/– Mice With Periodontitis. Front Cell Infect Microbiol (2020) 10(498). doi: 10.3389/fcimb.2020.00498 PMC7536370 33072621

[55] Mizraji G Segev H Wilensky A Hovav A-H. Isolation, Processing and Analysis of Murine Gingival Cells. J Vis Exp JoVE (2013) 77):e50388–8. doi: 10.3791/50388 PMC3731175 23851361

[56] Kelly BJ Gross R Bittinger K Sherrill-Mix S Lewis JD Collman RG. Power and Sample-Size Estimation for Microbiome Studies Using Pairwise Distances and PERMANOVA. Bioinformatics (2015) 31(15):2461–8. doi: 10.1093/bioinformatics/btv183 PMC4514928 25819674

[57] Segata N Izard J Waldron L Gevers D Miropolsky L Garrett WS. Metagenomic Biomarker Discovery and Explanation. Genome Biol (2011) 12(6):R60. doi: 10.1186/gb-2011-12-6-r60 21702898 PMC3218848

[58] Pinto SM Kim H Subbannayya Y Giambelluca MS Bösl K Ryan L. Comparative Proteomic Analysis Reveals Varying Impact on Immune Responses in Phorbol 12-Myristate-13-Acetate-Mediated THP-1 Monocyte-to-Macrophage Differentiation. Front Immunol (2021) 12:679458. doi: 10.3389/fimmu.2021.679458 34234780 PMC8255674

[59] Genin M Clement F Fattaccioli A Raes M Michiels C. M1 and M2 Macrophages Derived From THP-1 Cells Differentially Modulate the Response of Cancer Cells to Etoposide. BMC Cancer (2015) 15(1):577. doi: 10.1186/s12885-015-1546-9 26253167 PMC4545815

[60] Han YW. Fusobacterium Nucleatum: A Commensal-Turned Pathogen. Curr Opin Microbiol (2015) 23:141–7. doi: 10.1016/j.mib.2014.11.013 PMC4323942 25576662

[61] Olsen I Taubman MA Singhrao SK. Porphyromonas Gingivalis Suppresses Adaptive Immunity in Periodontitis, Atherosclerosis, and Alzheimer’s Disease. J Oral Microbiol (2016) 8:33029. doi: 10.3402/jom.v8.33029 27882863 PMC5122233

[62] Brennan CA Garrett WS. Fusobacterium Nucleatum - Symbiont, Opportunist and Oncobacterium. Nat Rev Microbiol (2019) 17(3):156–66. doi: 10.1038/s41579-018-0129-6 PMC6589823 30546113

[63] Sharma A. Persistence of Tannerella Forsythia and Fusobacterium Nucleatum in Dental Plaque: A Strategic Alliance. Curr Oral Health Rep (2020) 7(1):22–8. doi: 10.1007/s40496-020-00254-6 PMC9917731 36779221

[64] Li Y Mooney EC Xia X-J Gupta N Sahingur SE. A20 Restricts Inflammatory Response and Desensitizes Gingival Keratinocytes to Apoptosis. Front Immunol (2020) 11(365). doi: 10.3389/fimmu.2020.00365 PMC7078700 32218782

[65] Malcomson B Wilson H Veglia E Thillaiyampalam G Barsden R Donegan S. Connectivity Mapping (Sscmap) to Predict A20-Inducing Drugs and Their Antiinflammatory Action in Cystic Fibrosis. Proc Natl Acad Sci USA (2016) 113(26):E3725–34. doi: 10.1073/pnas.1520289113 PMC4932930 27286825

[66] Eke PI Dye BA Wei L Slade GD Thornton-Evans GO Borgnakke WS. Update on Prevalence of Periodontitis in Adults in the United States: NHANES 2009 to 2012. J Periodontol (2015) 86(5):611–22. doi: 10.1902/jop.2015.140520 PMC4460825 25688694

[67] Tonetti MS Jepsen S Jin L Otomo-Corgel J. Impact of the Global Burden of Periodontal Diseases on Health, Nutrition and Wellbeing of Mankind: A Call for Global Action. J Clin Periodontol (2017) 44(5):456–62. doi: 10.1111/jcpe.12732 28419559

[68] Wu YY Xiao E Graves DT. Diabetes Mellitus Related Bone Metabolism and Periodontal Disease. Int J Oral Sci (2015) 7(2):63–72. doi: 10.1038/ijos.2015.2 25857702 PMC4817554

[69] Peacock ME Arce RM Cutler CW. Periodontal and Other Oral Manifestations of Immunodeficiency Diseases. Oral Dis (2017) 23(7):866–88. doi: 10.1111/odi.12584 PMC5352551 27630012

[70] Ebersole JL Graves CL Gonzalez OA Dawson D III Morford LA Huja PE. Aging, Inflammation, Immunity and Periodontal Disease. Periodontol 2000 (2016) 72(1):54–75. doi: 10.1111/prd.12135 27501491

[71] Persson GR. Periodontal Complications With Age. Periodontol 2000 (2018) 78(1):185–94. doi: 10.1111/prd.12227 30198125

[72] Balta MG Papathanasiou E Blix IJ Van Dyke TE. Host Modulation and Treatment of Periodontal Disease. J Dent Res (2021) 100(8):798–809. doi: 10.1177/0022034521995157 33655803 PMC8261853

[73] Mizraji G Heyman O Van Dyke TE Wilensky A. Resolvin D2 Restrains Th1 Immunity and Prevents Alveolar Bone Loss in Murine Periodontitis. Front Immunol (2018) 9(785). doi: 10.3389/fimmu.2018.00785 PMC5996935 29922275

[74] Rich GT Buchweitz M Winterbone MS Kroon PA Wilde PJ. Towards an Understanding of the Low Bioavailability of Quercetin: A Study of Its Interaction With Intestinal Lipids. Nutrients (2017) 9(2):111. doi: 10.3390/nu9020111 PMC5331542 28165426

[75] Sadeghi-Ghadi Z Ebrahimnejad P Talebpour Amiri F Nokhodchi A. Improved Oral Delivery of Quercetin With Hyaluronic Acid Containing Niosomes as a Promising Formulation. J Drug Target (2021) 29(2):225–34. doi: 10.1080/1061186X.2020.1830408 32997536

[76] Cheng W-C Huang R-Y Chiang C-Y Chen J-K Liu C-H Chu C-L. Ameliorative Effect of Quercetin on the Destruction Caused by Experimental Periodontitis in Rats. J Periodontal Res (2010) 45(6):788–95. doi: 10.1111/j.1600-0765.2010.01301.x 20663021

[77] Napimoga MH Clemente-Napimoga JT Macedo CG Freitas FF Stipp RN Pinho-Ribeiro FA. Quercetin Inhibits Inflammatory Bone Resorption in a Mouse Periodontitis Model. J Natural Products (2013) 76(12):2316–21. doi: 10.1021/np400691n 24246038

[78] Wei Y Fu J Wu W Ma P Ren L Yi Z. Quercetin Prevents Oxidative Stress-Induced Injury of Periodontal Ligament Cells and Alveolar Bone Loss in Periodontitis. Drug Design Dev Ther (2021) 15:3509–22. doi: 10.2147/DDDT.S315249 PMC8366957 34408403

[79] Tang SM Deng XT Zhou J Li QP Ge XX Miao L. Pharmacological Basis and New Insights of Quercetin Action in Respect to its Anti-Cancer Effects. BioMed Pharmacother (2020) 121:109604. doi: 10.1016/j.biopha.2019.109604 31733570

[80] Lin R Piao M Song Y. Dietary Quercetin Increases Colonic Microbial Diversity and Attenuates Colitis Severity in Citrobacter Rodentium-Infected Mice. Front Microbiol (2019) 10:1092. doi: 10.3389/fmicb.2019.01092 31156598 PMC6531918

[81] Rinwa P Kumar A. Quercetin Suppress Microglial Neuroinflammatory Response and Induce Antidepressent-Like Effect in Olfactory Bulbectomized Rats. Neuroscience (2013) 255:86–98. doi: 10.1016/j.neuroscience.2013.09.044 24095694

[82] Patel RV Mistry BM Shinde SK Syed R Singh V Shin HS. Therapeutic Potential of Quercetin as a Cardiovascular Agent. Eur J Med Chem (2018) 155:889–904. doi: 10.1016/j.ejmech.2018.06.053 29966915

[83] Du G Zhao Z Chen Y Li Z Tian Y Liu Z. Quercetin Protects Rat Cortical Neurons Against Traumatic Brain Injury. Mol Med Rep (2018) 17(6):7859–65. doi: 10.3892/mmr.2018.8801 29620218

[84] Wang Q Liu C. Protective Effects of Quercetin Against Brain Injury in a Rat Model of Lipopolysaccharide-Induced Fetal Brain Injury. Int J Dev Neurosci (2018) 71:175–80. doi: 10.1016/j.ijdevneu.2018.09.008 30282008

[85] Khan A Ali T Rehman SU Khan MS Alam SI Ikram M. Neuroprotective Effect of Quercetin Against the Detrimental Effects of LPS in the Adult Mouse Brain. Front Pharmacol (2018) 9(1383). doi: 10.3389/fphar.2018.01383 PMC6297180 30618732

[86] Costa LG Garrick JM Roquè PJ Pellacani C. Mechanisms of Neuroprotection by Quercetin: Counteracting Oxidative Stress and More. Oxid Med Cell Longevity (2016) 2016:2986796–2986796. doi: 10.1155/2016/2986796 PMC4745323 26904161

[87] Haleagrahara N Miranda-Hernandez S Alim MA Hayes L Bird G Ketheesan N. Therapeutic Effect of Quercetin in Collagen-Induced Arthritis. Biomed Pharmacother (2017) 90:38–46. doi: 10.1016/j.biopha.2017.03.026 28342364

[88] Guazelli CFS Staurengo-Ferrari L Zarpelon AC Pinho-Ribeiro FA Ruiz-Miyazawa KW Vicentini F. Quercetin Attenuates Zymosan-Induced Arthritis in Mice. BioMed Pharmacother (2018) 102:175–84. doi: 10.1016/j.biopha.2018.03.057 29554596

[89] Rogerio AP Dora CL Andrade EL Chaves JS Silva LF Lemos-Senna E. Anti-Inflammatory Effect of Quercetin-Loaded Microemulsion in the Airways Allergic Inflammatory Model in Mice. Pharmacol Res (2010) 61(4):288–97. doi: 10.1016/j.phrs.2009.10.005 19892018

[90] Tabak C Arts ICW Smit HA Heederik D Kromhout D. Chronic Obstructive Pulmonary Disease and Intake of Catechins, Flavonols, and Flavones. Am J Respir Crit Care Med (2001) 164(1):61–4. doi: 10.1164/ajrccm.164.1.2010025 11435239

[91] Jafarinia M Sadat Hosseini M Kasiri N Fazel N Fathi F Ganjalikhani Hakemi M. Quercetin With the Potential Effect on Allergic Diseases. Allergy Asthma Clin Immunol (2020) 16:36. doi: 10.1186/s13223-020-00434-0 32467711 PMC7227109

[92] Townsend EA Emala CW Sr. Quercetin Acutely Relaxes Airway Smooth Muscle and Potentiates β-Agonist-Induced Relaxation via Dual Phosphodiesterase Inhibition of Plcβ and PDE4. Am J Physiol Lung Cell Mol Physiol (2013) 305(5):L396–403. doi: 10.1152/ajplung.00125.2013 PMC3763034 23873842

[93] Knekt P Kumpulainen J Järvinen R Rissanen H Heliövaara M Reunanen A. Flavonoid Intake and Risk of Chronic Diseases. Am J Clin Nutr (2002) 76(3):560–8. doi: 10.1093/ajcn/76.3.560 12198000

[94] Dhanya R Arya AD Nisha P Jayamurthy P. Quercetin, a Lead Compound Against Type 2 Diabetes Ameliorates Glucose Uptake via AMPK Pathway in Skeletal Muscle Cell Line. Front Pharmacol (2017) 8(336). doi: 10.3389/fphar.2017.00336 PMC5462925 28642704

[95] Mahmoud MF Hassan NA El Bassossy HM Fahmy A. Quercetin Protects Against Diabetes-Induced Exaggerated Vasoconstriction in Rats: Effect on Low Grade Inflammation. PloS One (2013) 8(5):e63784–4. doi: 10.1371/journal.pone.0063784 PMC3661670 23717483

[96] Salehi B Machin L Monzote L Sharifi-Rad J Ezzat SM Salem MA. Therapeutic Potential of Quercetin: New Insights and Perspectives for Human Health. ACS Omega (2020) 5(20):11849–72. doi: 10.1021/acsomega.0c01818 PMC7254783 32478277

[97] Xiong G Ji W Wang F Zhang F Xue P Cheng M. Quercetin Inhibits Inflammatory Response Induced by LPS From Porphyromonas Gingivalis in Human Gingival Fibroblasts via Suppressing NF-κb Signaling Pathway. BioMed Res Int (2019) 2019:6282635–6282635. doi: 10.1155/2019/6282635 31531360 PMC6720363

[98] Orecchioni M Ghosheh Y Pramod AB Ley K. Macrophage Polarization: Different Gene Signatures in M1(LPS+) vs. Classically and M2(LPS–) vs. Alternatively Activated Macrophages. Front Immunol (1084) 2019:10. doi: 10.3389/fimmu.2019.01084 PMC6543837 31178859

[99] Mubarak RA Roberts N Mason RJ Alper S Chu HW. Comparison of Pro- and Anti-Inflammatory Responses in Paired Human Primary Airway Epithelial Cells and Alveolar Macrophages. Respir Res (2018) 19(1):126–6. doi: 10.1186/s12931-018-0825-9 PMC6020222 29940963

[100] Cheng S-C Huang W-C Pang J-HS Wu Y-H Cheng C-Y. Quercetin Inhibits the Production of IL-1β-Induced Inflammatory Cytokines and Chemokines in ARPE-19 Cells via the MAPK and NF-κb Signaling Pathways. Int J Mol Sci (2019) 20(12):2957. doi: 10.3390/ijms20122957 PMC6628093 31212975

[101] Fuhrmann-Stroissnigg H Ling YY Zhao J McGowan SJ Zhu Y Brooks RW. Identification of HSP90 Inhibitors as a Novel Class of Senolytics. Nat Commun (2017) 8(1):422. doi: 10.1038/s41467-017-00314-z 28871086 PMC5583353

[102] Hasima N Ozpolat B. Regulation of Autophagy by Polyphenolic Compounds as a Potential Therapeutic Strategy for Cancer. Cell Death Dis (2014) 5(11):e1509–9. doi: 10.1038/cddis.2014.467 PMC4260725 25375374

[103] Jiang W Huang Y Han N He F Li M Bian Z. Quercetin Suppresses NLRP3 Inflammasome Activation and Attenuates Histopathology in a Rat Model of Spinal Cord Injury. Spinal Cord (2016) 54(8):592–6. doi: 10.1038/sc.2015.227 26754474

[104] Iske J Seyda M Heinbokel T Maenosono R Minami K Nian Y. Senolytics Prevent Mt-DNA-Induced Inflammation and Promote the Survival of Aged Organs Following Transplantation. Nat Commun (2020) 11(1):4289. doi: 10.1038/s41467-020-18039-x 32855397 PMC7453018

[105] Shin EJ Lee JS Hong S Lim T-G Byun S. Quercetin Directly Targets JAK2 and Pkcδ and Prevents UV-Induced Photoaging in Human Skin. Int J Mol Sci (2019) 20(21):5262. doi: 10.3390/ijms20215262 PMC6862686 31652815

[106] Khan F Niaz K Maqbool F Ismail Hassan F Abdollahi M Nagulapalli Venkata KC. Molecular Targets Underlying the Anticancer Effects of Quercetin: An Update. Nutrients (2016) 8(9):529. doi: 10.3390/nu8090529 PMC5037516 27589790

[107] Ruiz PA Braune A Hölzlwimmer G Quintanilla-Fend L Haller D. Quercetin Inhibits TNF-Induced NF-κb Transcription Factor Recruitment to Proinflammatory Gene Promoters in Murine Intestinal Epithelial Cells. J Nutr (2007) 137(5):1208–15. doi: 10.1093/jn/137.5.1208 17449583

[108] Zhang W Jia L Zhao B Xiong Y Wang Y-N Liang J. Quercetin Reverses TNF−α Induced Osteogenic Damage to Human Periodontal Ligament Stem Cells by Suppressing the NF−κb/NLRP3 Inflammasome Pathway. Int J Mol Med (2021) 47(4):39. doi: 10.3892/ijmm.2021.4872 33537804 PMC7891819

[109] Xiao X Shi D Liu L Wang J Xie X Kang T. Quercetin Suppresses Cyclooxygenase-2 Expression and Angiogenesis Through Inactivation of P300 Signaling. PloS One (2011) 6(8):e22934–4. doi: 10.1371/journal.pone.0022934 PMC3152552 21857970

[110] Ren K-W Li Y-H Wu G Ren J-Z Lu H-B Li Z-M. Quercetin Nanoparticles Display Antitumor Activity via Proliferation Inhibition and Apoptosis Induction in Liver Cancer Cells. Int J Oncol (2017) 50(4):1299–311. doi: 10.3892/ijo.2017.3886 28259895

[111] Martens A van Loo G. A20 at the Crossroads of Cell Death, Inflammation, and Autoimmunity. Cold Spring Harb Perspect Biol (2020) 12(1):a036418. doi: 10.1101/cshperspect.a036418 31427375 PMC6942121

[112] Carlos-Reyes Á López-González JS Meneses-Flores M Gallardo-Rincón D Ruíz-García E Marchat LA. Dietary Compounds as Epigenetic Modulating Agents in Cancer. Front Genet (2019) 10(79). doi: 10.3389/fgene.2019.00079 PMC6406035 30881375

[113] Atrahimovich D Samson AO Barsheshet Y Vaya J Khatib S Reuveni E. Genome-Wide Localization of the Polyphenol Quercetin in Human Monocytes. BMC Genomics (2019) 20(1):606. doi: 10.1186/s12864-019-5966-9 31337340 PMC6652105

[114] Akbari Kordkheyli V Khonakdar Tarsi A Mishan MA Tafazoli A Bardania H Zarpou S. Effects of Quercetin on microRNAs: A Mechanistic Review. J Cell Biochem (2019) 120(8):12141–55. doi: 10.1002/jcb.28663 30957271

[115] Wang F Ke Y Yang L Wang FJ. Quercetin Protects Human Oral Keratinocytes From Lipopolysaccharide-Induced Injury by Downregulating microRNA-22. Hum Exp Toxicol (2020) 39(10):1310–7. doi: 10.1177/0960327120918291 32329368

[116] Zhu B Macleod LC Newsome E Liu J Xu P. Aggregatibacter Actinomycetemcomitans Mediates Protection of Porphyromonas Gingivalis From Streptococcus Sanguinis Hydrogen Peroxide Production in Multi-Species Biofilms. Sci Rep (2019) 9(1):4944. doi: 10.1038/s41598-019-41467-9 30894650 PMC6426879

[117] Vander Haar EL So J Gyamfi-Bannerman C Han YW. Fusobacterium Nucleatum and Adverse Pregnancy Outcomes: Epidemiological and Mechanistic Evidence. Anaerobe (2018) 50:55–9. doi: 10.1016/j.anaerobe.2018.01.008 PMC6750227 29409815

[118] Nie J Zhang L Zhao G Du X. Quercetin Reduces Atherosclerotic Lesions by Altering the Gut Microbiota and Reducing Atherogenic Lipid Metabolites. J Appl Microbiol (2019) 127(6):1824–34. doi: 10.1111/jam.14441 31509634

[119] Tan S Caparros-Martin JA Matthews VB Koch H O’Gara F Croft KD. Isoquercetin and Inulin Synergistically Modulate the Gut Microbiome to Prevent Development of the Metabolic Syndrome in Mice Fed a High Fat Diet. Sci Rep (2018) 8(1):10100. doi: 10.1038/s41598-018-28521-8 29973701 PMC6031638

[120] Porras D Nistal E Martinez-Florez S Pisonero-Vaquero S Olcoz JL Jover R. Protective Effect of Quercetin on High-Fat Diet-Induced non-Alcoholic Fatty Liver Disease in Mice is Mediated by Modulating Intestinal Microbiota Imbalance and Related Gut-Liver Axis Activation. Free Radic Biol Med (2017) 102:188–202. doi: 10.1016/j.freeradbiomed.2016.11.037 27890642

[121] Xie J Song W Liang X Zhang Q Shi Y Liu W. Protective Effect of Quercetin on Streptozotocin-Induced Diabetic Peripheral Neuropathy Rats Through Modulating Gut Microbiota and Reactive Oxygen Species Level. BioMed Pharmacother (2020) 127:110147. doi: 10.1016/j.biopha.2020.110147 32559841

[122] Han MK Barreto TA Martinez FJ Comstock AT Sajjan US. Randomised Clinical Trial to Determine the Safety of Quercetin Supplementation in Patients With Chronic Obstructive Pulmonary Disease. BMJ Open Respir Res (2020) 7(1):e000392. doi: 10.1136/bmjresp-2018-000392 PMC7047491 32071149

[123] Lu NT Crespi CM Liu NM Vu JQ Ahmadieh Y Wu S. A Phase I Dose Escalation Study Demonstrates Quercetin Safety and Explores Potential for Bioflavonoid Antivirals in Patients With Chronic Hepatitis C. Phytother Res PTR (2016) 30(1):160–8. doi: 10.1002/ptr.5518 PMC5590840 26621580

[124] Basu A Masek E Ebersole JL. Dietary Polyphenols and Periodontitis-A Mini-Review of Literature. Molecules (2018) 23(7):1786. doi: 10.3390/molecules23071786 PMC6099717 30036945

[125] Dabeek WM Marra MV. Dietary Quercetin and Kaempferol: Bioavailability and Potential Cardiovascular-Related Bioactivity in Humans. Nutrients (2019) 11(10):2288. doi: 10.3390/nu11102288 PMC6835347 31557798

[126] Wang Y Tao B Wan Y Sun Y Wang L Sun J. Drug Delivery Based Pharmacological Enhancement and Current Insights of Quercetin With Therapeutic Potential Against Oral Diseases. Biomed Pharmacother (2020) 128:110372. doi: 10.1016/j.biopha.2020.110372 32521458

